# The impacts of peace and conflict resolution interventions on mental health and psychological well-being: a systematic review

**DOI:** 10.1186/s13031-026-00804-2

**Published:** 2026-05-31

**Authors:** María Camila García Durán, Rakesh Singh, Mauricio Avendano, Crick Lund

**Affiliations:** 1 Centre for Global Mental Health, Health Service and Population Research Department, Institute of Psychiatry, Psychology and Neuroscience, King’s College London, Bogotá, Colombia; 2 Salud Poblacional, Fundación Santa Fe de Bogotá, Bogotá, Colombia; 3 Research Department, Transcultural Psychosocial Organization (TPO) Nepal, Kathmandu, Nepal; 4 Center for Primary Care and Public Health (Unisanté), University of Lausanne, Lausanne, Switzerland; 5 Department of Social and Behavioral Sciences, Harvard T.H. Chan School of Public health, Boston, USA; 6 Department of Psychiatry and Mental Health, Alan J Flisher Centre for Public Mental Health, University of Cape Town, Cape Town, South Africa

**Keywords:** Armed conflict, Mental health, Conflict resolution, Peace

## Abstract

**Background:**

Prior studies have examined the impact of armed conflict on mental health. This systematic review synthesizes evidence on how peace and conflict resolution interventions influence the mental health and well-being of conflict-affected communities.

**Methods:**

We conducted a systematic review across multiple databases and identified studies evaluating the effects of peace and conflict resolution interventions on mental health and well-being from 1990 to 2026. Due to heterogeneity in study designs and outcomes, a narrative synthesis was conducted.

**Results:**

The search yielded 6,360 records, of which 15 were included. The majority of studies were cross-sectional (*n* = 6), and only two were randomised controlled trials (RCTs). Most studies were conducted in African countries, targeted the community level, and were implemented by government agencies or non-governmental organisations (NGOs). Overall, studies yield mixed mental health outcomes. On the one hand, rehabilitation programmes, reparations support, financial aid, and reconciliation media show positive effects on psychological well-being. However, truth and reconciliation interventions were associated with negative outcomes, as they exacerbate distress, posttraumatic stress disorder, and anxiety, particularly for survivors. Most studies were limited by a focus on short-term effects and lack of a comparison group.

**Conclusion:**

The findings present a mixed picture: while rehabilitation and economic interventions show mental health benefits, truth and reconciliation efforts were associated with psychological harms. More longitudinal studies with larger samples and rigorous designs are needed to assess long-term effects. Recognising both the mental health risks and benefits of peace and conflict interventions is essential to ensure they support not only political stability but also psychological recovery and resilience.

**Supplementary Information:**

The online version contains supplementary material available at 10.1186/s13031-026-00804-2.

## Background

The Stockholm International Peace Research Institute [1] defines armed conflict as the use of armed force between two or more states or non-state organised armed groups, resulting in at least 25 battle-related deaths within a year. In recent years, global security has deteriorated, with an estimated increase in the number of conflict-related deaths worldwide from 131,752 in 2015 to 154,626 in 2023 [2]. Between 2020 and 2023, some of the highest fatality rates since the Rwandan genocide in 1994 were recorded, and in 2023 the number of active state-based armed conflicts reached 59, the highest ever recorded by the Uppsala Conflict Data Program [3]. The devastating consequences of ongoing conflicts in Ukraine, Palestine, Sudan, the Democratic Republic of Congo, and Myanmar further highlight the urgency of understanding how peace and conflict resolution interventions impact psychological resilience and recovery in affected populations [4–8].

Beyond mortality, armed conflicts have far-reaching consequences for surviving communities, affecting population health [9–13], disrupting education systems [14, 15], hindering economic growth [16, 17], and eroding the social fabric [18, 19]. Among the lasting scars of armed conflict are its profound impacts on mental health [20–23], with individuals exposed to armed conflict facing high prevalence rates of anxiety, depression and post-traumatic stress disorder (PTSD) that are two to three times higher than the rate for unexposed individuals, with women and children being particularly vulnerable [24].

While systematic reviews have summarised the impact of armed conflict on mental health, and the effects of psychosocial support programmes specifically designed to protect and promote mental health in crisis-affected populations [25–28], no review has synthesised evidence on how peace and conflict resolution interventions affect mental health and well-being. This gap is significant: peace interventions are increasingly deployed in post-conflict settings, yet their psychological consequences, whether beneficial or harmful, remain poorly understood.

Recognition of this gap has grown considerably at the policy level in recent years. The United Nations Development Programme (UNDP) [29] and the Inter-Agency Standing Committee (IASC) [30] have both published guidance calling for a bidirectional integration of mental health and psychosocial support (MHPSS) and peacebuilding, noting that peace cannot be sustainable if the psychological impacts of conflict on affected communities remain unaddressed. Despite this growing policy consensus, the empirical evidence base underpinning this integration remains limited, reinforcing the need for systematic synthesis of what is known about the mental health effects of peace interventions.

Conflicts are not static but evolve through phases of escalation and de-escalation, moving from active wars toward unstable and ultimately stable peace [31, 32]. Different types of interventions may be implemented across these phases to address political violence and its consequences [33]. This review focuses specifically on interventions implemented during the de-escalation and post-conflict phases, defined as structured initiatives aimed at resolving political conflict, preventing relapse into violence, and promoting societal recovery [33]. These include peacekeeping, peacebuilding, and peace consolidation efforts such as truth and reconciliation commissions, reparations programmes, community-based justice mechanisms, economic reintegration initiatives, and peace agreements.

Post-conflict interventions are essential for sustainable peace because the risk of relapse into war is high in the immediate post-conflict period [31–34]. Evaluating both the potential benefits and risks of these interventions on mental health is therefore crucial for designing peacebuilding processes that prevent a return to violence and promote long-term psychological recovery.

This paper reports the results of a systematic review of the literature on how peace interventions during the de-escalation phase affect the mental health and well-being of surviving communities in post-conflict settings. Specifically, it addresses the following research question: How do peace interventions impact the mental health and well-being of individuals in post-conflict contexts? Following Patel et al.’s definition of mental health [35], we assess a broad range of outcomes, including clinical diagnoses or symptoms, and the ability to cope with life stresses, work productively, contribute to communities, and reach one’s full potential. To our knowledge, this is the first systematic review of how peace and conflict resolution interventions impact the mental health and well-being of surviving communities.

## Methods

The Preferred Reporting Items for Systematic Reviews and Meta-Analyses (PRISMA) guidelines [36] were followed to ensure transparency and rigor in study selection, data extraction, and synthesis. Inclusion criteria were: (a) quantitative studies with observational (cross-sectional, cohort) or experimental (randomised controlled trials - RCTs, quasi-experimental) designs; (b) studies reporting changes in or effects on mental health and well-being from peace programs or interventions; (c) publications in any language with an abstract in English; (d) studies involving human subjects; and (e) studies published between January 1, 1990, and April 9, 2026. No age restrictions were applied.

Exclusion criteria included: (a) interventions consisting solely of psychological components (e.g., cognitive behavioural therapy (CBT), mindfulness) without peace-related elements; (b) peace interventions unrelated to armed conflict or in post-conflict settings; and (c) studies that did not quantify mental health outcomes (e.g., qualitative-only designs) as the review aimed to synthesise evidence on the direction and magnitude of effects across studies using a common metric. Reviews, commentaries, books, conference presentations, posters, case studies, and grey literature were also excluded, to ensure methodological consistency and reproducibility of the search, though reviews were screened for eligible studies.

A systematic search was conducted across multiple databases, including PsycINFO, MEDLINE, Embase, Scopus, PTDSpubs (formerly PILOTS), LILACS (Latin American and Caribbean Health Sciences Literature), JSTOR, and EconLit. Search terms were structured around four concepts: (a) armed conflict, (b) peace interventions, (c) mental health outcomes, and (d) conflict-affected countries. Full search strategies are available in the Appendix A. Eligible papers were imported into EndNote, and duplicates were removed. Remaining records were screened in Rayyan [37], where a second duplicate check was performed. Two reviewers (MCGD and RS) screened titles, abstracts, and full texts. Disagreements were resolved through discussion with a third reviewer (CL or MA). Key data were extracted by the primary author and reviewed by a second reviewer, with inconsistencies resolved through discussion with a third reviewer when needed.

Risk of bias was assessed independently by two reviewers (MCGD and RS). The Joanna Briggs Institute (JBI) Critical Appraisal Checklist for Analytical Cross-Sectional Studies was utilized for evaluating cross-sectional articles. For cohort and quasi-experimental designs, the Cochrane Collaboration’s ROBINS-I tool was employed, while the Cochrane Risk-of-Bias Tool for Randomized Trials (RoB 2) was used for randomized trials.

All studies included in the review were analysed using narrative synthesis. Studies were categorised using an adapted version of the socio-ecological framework [38] as a guide to organising and summarising the evidence according to the level at which the intervention is intended to operate. Intervention levels were categorised as individual, family (household), community (including intergroup interventions) or national. A meta-analysis was not performed because of the considerable methodological heterogeneity of publications.

The review protocol was registered with PROSPERO on 3 January 2024 (CRD42024494800). Although the protocol limited inclusion to studies using validated instruments, this criterion was removed during screening as many studies did not report on validation.

## Results

**Fig. 1:**
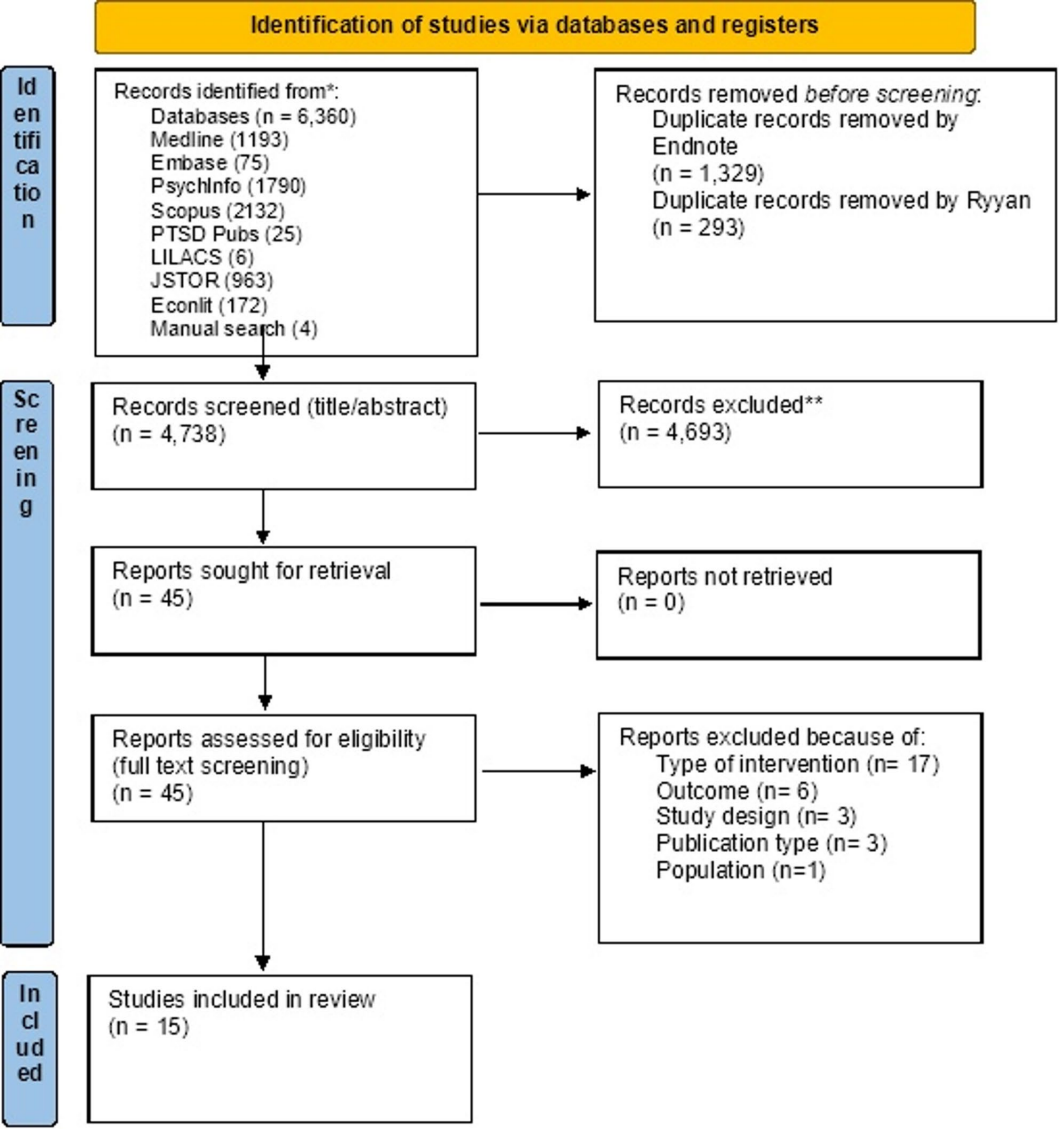
PRISMA flow-chart (study selection)

Figure 1 summarizes the study identification and selection process. The initial search yielded 6,360 records, with 1,622 duplicates removed. Titles and abstracts of 4,738 papers were screened, and 45 full texts reviewed. Fifteen studies were included in the final review.

Table 1 summarizes the sample characteristics and outcome measures of the included studies. A range of mental health outcomes were reported, being PTSD the most common (*n* = 8, 53%), followed by depression (*n* = 6, 40%) and anxiety (*n* = 3, 20%). Other non-clinical outcomes included intensity of primary emotions, individual negative emotions, neuroticism, self-efficacy, and self-esteem.

**Table 1:** Overview of studies included in the review

First author (year), country	Brief description of the intervention	Design	Sample size	Age (M= mean age; SD= Standard deviation)	Sex	Outcome measures (instrument)
Individual-level interventions						
Adhikari et al. (2015), Nepal	**Rehabilitation package for former Nepali combatants**: included four types of support: formal education support, financing a micro-enterprise, vocational skill training and training in the health sector.	Longitudinal study	Baseline: 400 Follow-up: 310	Age range: 16–23; M = 20.6; SD = 1.4	Male (63.9%), Female (36.1%)	Anxiety, depression (*Hopkins Symptoms* *Checklist - HSCL)* and posttraumatic stress disorder (*PTSD Checklist-civilian version- PCL-C)*
**Family (household)-level interventions**						
Leon-Jurado et al. (2022), Colombia	**Transformando mi Futuro**: aimed to equip victims of the Colombian armed conflict with tools to optimize the use of their monetary reparation compensations.	Pre-post design (without control group)	Baseline: 531 households (549 participants) Endline: 464 households (474 participants) An average of two children and two women integrated the beneficiary households	Household mean age: M = 32	Female (74%)	Perception of wellbeing (*“Ladder of life”*)
Glass et al. (2017), Rural Eastern Democratic Republic of Congo (DRC)	**“Pigs for Peace” (PFP)**: hybrid program combining microcredit and productive asset transfer principles in rural Democratic Republic of Congo.	Randomized controlled trial	Intervention group: 309 Control group 1: 296 Control group 2: 228	Age range: 15–60. Most participants (78%) were 25 years or older	Intervention: Female (81.5%) Control: Female (86%)	Anxiety, depression (*HSCL*) and posttraumatic stress disorder (*adapted version of the Harvard Trauma Questionnaire - HTQ*)
**Community-level interventions**						
Kanyangara et al. (2007), Rwanda	**Gacaca courts in Rwanda**: consisted of 12,000 tribunals spread across all communities. Accused individuals presented their actions before a judge, survivors, and the community. The involved parties were encouraged to hear each other’s grievances and suffering to promote understanding and empathy.	Pre-post design (without control group)	*N* = 100 50 accused prisoners and 50 survivors	Survivors: Age range = 18–53; M = 31.7; SD = 8.8 Prisoners: Age range: 28–54; M = 37.0; SD = 6.8	Survivors: Female (68%) Prisoners: Male (84%)	Intensity of primary emotions (sadness/grief, anger, fear, disgust, anxiety, shame and guilt) (*shortened version of Izard’s Differential* *Emotions Scale*)
Brounéus (2010), Rwanda		Cross-sectional (comparing witnesses vs. non-witnesses)	*N* = 1,200	Age groups: Most participants were 35–44 (31%)	Female (51%)	Posttraumatic Stress Disorder (*PCL-C*), Depression (*HSCL*)
Caparos et al. (2020), Rwanda		Cross-sectional (comparing those who had never attended the Gacaca, those who had attended without testifying and those who had attended and testified)	*N* = 679	Age range: 27–64 years; M = 37; SD = 7.4	Female (50.35%)	Posttraumatic Stress Disorder (*PCL-C*), Depression (*Hopkins Depression* *Symptom Checklist – HDSC*)
Rimé et al. (2011), Rwanda		Pre-post design (without control group)	Experimental group: 384 (200 victims and 184 perpetrators) Control group: 371 (195 victims and 176 perpetrators)	Experimental group: - Victims: M = 30.60; SD = 7.70 - Perpetrators: M = 42.70; SD = 7.83 Control group: - Victims: M = 28.89; SD = 6.54 - Perpetrators: M = 44.31; SD = 7.78	Victims: Female (60.25%) Perpetrators: Female (45%)	Individual Negative Emotion (*Likert scale*), Post-Traumatic Stress Disorder (*Likert scale*)
Cilliers et al. (2016), Sierra Leone	**Community-based truth telling program in Sierra Leone**: Program which involved training community committees in trauma healing and mediation and encouraging both victims and perpetrators to engage in a community-based truth-telling process.	Randomized controlled trial	Wave 1 Baseline: 952 Endline: 885 Follow up: 850 Wave 2 Baseline 1430 Endline: 1273	Adults. Age not specified	Not specified	Anxiety, depression (*Zung Depression and Zung Anxiety* *Indices)* and posttraumatic stress disorder (*PTSD Symptom Scale*)
Kang et al. (2020), Rwanda	**Cows for Peace (CFP)**: aimed to foster reconciliation between genocide perpetrators and survivors through three key components: a 3-day cognitive-behavioural workshop to address post-traumatic stress, monthly self-led cell groups for ongoing support, and a cooperative cow-raising initiative that encouraged collaboration and shared benefits between the dyads.	Longitudinal study	*N* = 91 46 genocidaires and 45 survivors	Survivors: M = 59.8 Genocidaires: M = 56.4	Survivors: Female (51.1%) Genocidaires: Male (100%)	Posttraumatic stress disorder (*5-point Likert scale*)
Weder et al. (2010), Israel-Palestine	**The Parents Circle-Families Forum (PCFF)**: cross-community peace-building project that included a telephone service for one-on-one conversations between Israelis and Palestinians, a school speaker program, and community lectures.	Cross-sectional (comparing intervention participants vs. non-participants)	*N* = 21 13 members of Parents Circle Family Forum (PC group) and 8 non-PC group	PC group: M = 44.33 Non-PC group: M = 45.75	PC group: Female (38.46%) Non-PC group: Female (75%)	Depression (*Beck Depression Inventory – BDI*); Prolonged grief disorder (*Prolonged Grief Disorder Scale - PG-13*); Distress (*Global Severity Index of Symptoms Checklist 90 - SCL-90-R*)
**National-level interventions**						
Stein et al. (2008), South Africa	**South African Truth and Reconciliation Commission (TRC)**: aimed to uncover and record apartheid-era human rights abuses, with survivors sharing their testimonies privately or publicly.	Cross-sectional (comparing those who participated in the TRC either providing testimony or information, attending public meetings, or watching/listening to the TRC hearings on television or radio to those who did not share information or participate in the TRC)	*N* = 4351	Not specified	Not specified	Distress (*30-day nonspecific distress scale*), anger (*Likert scale*)
Kaminer et al. (2001), South Africa		Cross-sectional (comparing those who had testified at a public hearing of the TRC, those who had given a closed statement to a TRC statement-taker, and those who did not give a statement or public testimony)	*N* = 134	Age range: 25–86; M = 53; SD = 14.3	Female (53.7%)	Anxiety, depression (*Mini-International Neuropsychiatric Interview*) and posttraumatic stress disorder (*Composite International Diagnostic Interview’s PTSD event module*)
Simancas-Fernandez et al. (2022), Colombia	**Colombian Peace Accord reparations**: The study involved as victims of the armed conflict who had received reparations under Colombian Law 1448, 2011. The law mandated reparations through restitution, compensation, rehabilitation, satisfaction, and guarantees of non-repetition, addressing various dimensions of harm, including individual, collective, material, moral, and symbolic aspects. The impact of these reparations on quality of life was assessed.	Cross-sectional (All participants had received some type of repairment action. Comparisons were made between sociodemographic variables, type of aid received, and type of experienced traumatic event).	*N* = 1,139	Age range = 16–85; M = 42.51; SD = 15.39	Female (71%)	Quality of life, specifically, satisfaction with mental health (*WHOQOL-BREF*)
Qouta et al. (1995), Israel-Palestine	**Israel and Palestine peace treaty**: The aim was to explore the impact of the peace treaty between Israel and Palestine, signed in Washington on 13 September 1993, on the psychological well-being of Palestinian children.	Pre-post design (without control group)	Baseline: 108 Endline: 64	Participants were 11–12 years of age when tested, and 1 year older when retested	Girls (Baseline 53/Endline 35) and boys (Baseline 55/Endline 29) Baseline: Male (51%) Endline: Male (45%)	Neuroticism (*Eysenck neuroticism scale*), Self-esteem (*Rosenberg’s scale*)
Best et al. (2011), Liberia	**Media intervention for Truth and Reconciliation Commission**: Digital intervention aimed to examine the role of media in facilitating truth-telling and intra-psychic healing. Participants engaged with two media systems and contributed to content related to conflict and reconciliation in Liberia.	Pre-post design (with control group)	Treatment group: 72 Control group: 61	Ages ranged from 16 to 50 with just over half of the subjects falling within the 21–25 age range	Male (94%)	Self-efficacy (*General Self-Efficacy Inventory*)

The most frequent study designs were cross-sectional (*n* = 6, 40%) and pre-post (*n* = 5, 33%) studies. Only 2 studies (13%) were RCTs. Most studies (*n* = 10, 67%) were conducted in African countries, mainly Rwanda (*n* = 5, 33%) and South Africa (*n* = 2, 13%). Participants were typically adults (*n* = 12, 80%) or individuals over 16 (*n* = 2, 13%), with only one study including adolescents (ages 11–12). All studies included both female and male participants, though female participation was often higher. A brief description of each included intervention can be found in the Appendix B.

Interventions primarily targeted the community level (*n* = 7, 47%), followed by national (*n* = 5, 33%), family/household (*n* = 2, 13%), and individual (*n* = 1, 7%) levels. Government agencies were the main implementers (*n* = 9, 60%), followed by local NGOs and non-profits (*n* = 4, 27%). Only 27% (*n* = 4) [39–42] of studies reported adapting outcome measures (e.g., translation, pilot testing, or local validation). Most used validated instruments without mentioning local adaptation (*n* = 8, 53%) [43–50], while others provided insufficient detail.

Table 2 summarizes the main findings by mental health outcome. Intervention effects varied: seven studies (47%) reported improvements, four (27%) found negative changes, two (13%) showed mixed results, and two (13%) reported no statistically significant changes.

**Table 2:** Main findings for each intervention by mental health outcomes

First author (year), country	Intervention	Mental health outcomes			
		PTSD	Depression	Anxiety	Other mental health outcome
Individual-level interventions					
Adhikari et al. (2015), Nepal	**Rehabilitation package for former Nepali combatants**	Significant reduction from baseline to follow-up (β = −2.47, 95% CI: −4.37, −0.57, *p* = 0.01). PTSD prevalence dropped from 30% to 7.2% over the study period. *Gender by package interaction effect*: Males who participated in the health package (β = 11.31, 95% CI: 2.72, 19.91, *p* = 0.01) and those in vocational training (β = 9.31, 95% CI: 0.30, 18.31, *p* = 0.04) had significantly higher PTSD symptoms when compared to females and education groups, with scores increasing by approximately 11 and 9 points on the PCL-C scale (clinical cutoff = 50).	No significant change over time β = −0.40, 95% CI: −1.51, 0.71, *p* = 0.48). Depression prevalence dropped from 34.8% in baseline to 30% in follow-up. *Gender by package interaction effect*: Boys who participated in the health package had significantly higher depression symptoms (β = 8.44, 95% CI: 2.67, 14.21, *p* = 0.004), meaning their HSCL-25 depression scores were on average 8.44 points higher than those in female and education groups.	Significant reduction from baseline to follow-up (β = −0.76, 95% CI: −1.49, −0.04, *p* = 0.04). Prevalence dropped from 33.5% to 24.4% between baseline and follow-up. No significant gender by package interaction effect was found.	
**Family (household)-level interventions**					
Leon-Jurado et al. (2022), Colombia	**Transformando mi Futuro**				There was a significant increase in *perceived well-being* from baseline (M = 5.10) to endline (M = 5.48) (β = 0.381, *p* < 0.01), as measured on a 10-point well-being scale. Mean well-being scores increased from 5.10 at baseline to 5.48 at endline.
Glass et al. (2017), Rural Eastern Democratic Republic of Congo (DRC)	Pigs For Peace	There was a significant reduction in PTSD symptoms in the intervention group compared to the control group from baseline (M = 2.17, SD = 0.63) to 18 months (M = 1.75, SD = 0.48) (β = −0.137, 95% CI: −0.211, −0.062, *p* = 0.004), as measured by the Harvard Trauma Questionnaire (HTQ).	No significant difference in depression symptoms between the intervention and control groups from baseline (M = 1.77, SD = 0.46) to 18 months (M = 1.60, SD = 0.44) (β = −0.055, 95% CI: −0.119, 0.008, *p* = 0.089), as measured by the Hopkins Symptom Checklist (HSCL-25).	There was a significant reduction in anxiety symptoms in the intervention group compared to the control group from baseline (M = 1.70, SD = 0.54) to 18 months (M = 1.58, SD = 0.50) (β = −0.086, 95% CI: −0.159, −0.012, *p* = 0.023), as measured by the Hopkins Symptom Checklist (HSCL-25)	
**Community-level interventions**					
Kanyangara et al. (2007), Rwanda	**Gacaca courts in Rwanda**				The intensity of most *negative primary emotions* (sadness, fear, disgust, anxiety, and shame) significantly increased after participation in the gacaca tribunals, particularly among survivors. • Sadness: Increased from M = 3.46 (SD = 0.86) to M = 4.50 (SD = 0.86) among survivors, and from M = 3.76 (SD = 0.71) to M = 4.16 (SD = 0.93) among prisoners **(p < 0.01).** • Disgust: Increased from M = 2.88 (SD = 0.98) to M = 4.48 (SD = 1.16) among survivors, and from M = 3.56 (SD = 1.19) to M = 4.04 (SD = 1.24) among prisoners **(p < 0.01).** • Shame: Increased from M = 2.66 (SD = 0.96) to M = 4.26 (SD = 1.24) among survivors, and from M = 3.22 (SD = 1.14) to M = 3.96 (SD = 1.06) among prisoners **(p < 0.01).** • Anxiety: Increased from M = 2.40 (SD = 0.98) to M = 4.50 (SD = 0.97) among survivors, and from M = 3.00 (SD = 1.35) to M = 4.06 (SD = 1.20) among prisoners **(p < 0.01).** Fear increased among survivors from M = 2.98 (SD = 1.02) to M = 4.38 (SD = 1.10) (t(49) = −6.93, *p* < 0.01), and among prisoners from M = 3.10 (SD = 1.21) to M = 3.52 (SD = 1.21). This increase in fear was significantly greater for survivors than for prisoners (t(49) = −1.78, *p* = 0.09). Anger decreased among prisoners from M = 3.72 (SD = 0.94) to M = 3.12 (SD = 0.82) (**p < 0.01**), while it remained stable for survivors (M = 3.64, SD = 0.77 to M = 3.68, SD = 0.76), with the change being nonsignificant. Additionally, guilt significantly increased among prisoners from M = 2.72 (SD = 0.64) to M = 3.28 (SD = 0.88) **(p < 0.01**), while for survivors there was a nonsignificant decrease (M = 2.52, SD = 0.73 to M = 2.38, SD = 0.69).
Brounéus (2010), Rwanda		PTSD symptoms were significantly higher among survivors who had witnessed in the gacaca compared to those who had not (**RR = 1.40**,**p < 0.001**). Among inyangamugayo (judges) and neighbors, witnessing in the gacaca was associated with a 75% higher relative risk of PTSD (**RR = 1.75**,**p< 0.01**) compared to those who did not witness.	Depression symptoms were significantly higher among survivors who had witnessed in the gacaca compared to those who had not (**RR = 1.20**,**p < 0.05**). Among inyangamugayo (judges) and neighbors, witnessing in the gacaca was associated with a 60% higher relative risk of depression (**RR = 1.60**,**p < 0.01**).		
Caparos et al. (2020), Rwanda		PTSD symptoms were significantly lower among survivors who attended the Gacaca tribunals (M = 33.7, SD = 11.6) compared to those who testified (M = 45.5, SD = 14.1), t(76) = 5.7, *p* < 0.001, d = 0.94. Additionally, PTSD symptoms were lower in survivors who attended the Gacaca compared to those who did not (**p = 0.027**) and lower in neighbors who attended compared to those who did not (p = 0.096).	Depression symptoms were significantly higher among survivors than neighbors, F(1, 527) = 4.6, *p* = 0.031. Additionally, individuals who attended the Gacaca had lower depression symptoms than both those who did not attend (**p= 0.006**) and those who testified (**p = 0.087**). Those who suffered worse consequences from the genocide reported significantly higher depression symptoms, F(1, 527) = 44.7, *p* < 0.001		
Rimé et al. (2011), Rwanda		PTSD symptoms remained stable from the first to the second measurement in the control groups. However, among prisoners, PTSD symptoms decreased from M = 2.4 (SD = 0.56) to M = 2.1 (SD = 0.38), while among victims, PTSD symptoms increased significantly from M = 2.4 (SD = 0.47) to M = 3.31 (SD = 0.45) (**p < 0.001**)			Participation in the Gacaca tribunals was associated with an increase in *negative emotions* (fear, sadness, and anxiety) among both victims and perpetrators. • Victims: • Fear: Increased from M = 2.89 (SD = 1.34) to M = 4.81 (SD = 0.79) (**p < 0.001**). • Sadness: Increased from M = 4.06 (SD = 0.79) to M = 5.49 (SD = 1.71) (**p < 0.001**). • Anxiety: Increased from M = 1.77 (SD = 1.24) to M = 3.91 (SD = 0.83) (**p < 0.001**). • Perpetrators: • Fear: Increased from M = 3.34 (SD = 1.03) to M = 4.03 (SD = 0.99) (**p < 0.01**). • Sadness: Increased from M = 2.53 (SD = 0.82) to M = 3.63 (SD = 0.67) (**p < 0.001**). • Anxiety: Increased from M = 2.90 (SD = 1.21) to M = 3.68 (SD = 0.85) (**p < 0.001**).
Cilliers et al. (2016), Sierra Leone	**Community-based truth telling program in Sierra Leone**	PTSD symptoms significantly increased following participation in the reconciliation process (β = 0.029, *p* < 0.001). *Note: indicators are inverted; negative coefficients indicate worsening outcomes*	A significant increase in depression symptoms was observed following participation in the reconciliation process (β = −0.289, 95% CI not reported, *p* < 0.001). *Note: indicators are inverted; negative coefficients indicate worsening outcomes*	A significant increase in anxiety symptoms was observed following participation in the reconciliation process (β = −0.441, 95% CI not reported, *p* < 0.001). *Note: indicators are inverted; negative coefficients indicate worsening outcomes*	
Kang et al. (2020), Rwanda	**Cows for Peace (CFP)**	Survivors: PTSD symptoms decreased from M = 30.11 (SD = 5.68) at baseline to M = 19.34 (SD = 4.48) at follow-up (95% CI: −12.69, −8.80, *p* < 0.05). Génocidaires: PTSD symptoms decreased from M = 24.26 (SD = 6.32) to M = 16.09 (SD = 3.58) (95% CI: −12.69, −8.80, *p* < 0.05).			
Weder et al. (2010), Israel-Palestine	**The Parents Circle-Families Forum (PCFF)**		No significant differences in depression symptoms were found between groups.		Participants who did not belong to intervention group (PCFF) reported significantly higher *cognitive*,* emotional*,* and behavioural symptoms* (M = 0.63, SD = 0.183) compared to those who did (M = 0.17, SD = 0.112) (**p < 0.05**) No significant differences in *grief symptoms* or *psychological distress* were found between groups.
**National-level interventions**					
Stein et al. (2008), South Africa	**South African Truth and Reconciliation Commission (TRC)**				Attending the TRC was significantly associated with increased *psychological distress* (β = 0.198, *p* < 0.001), and individuals who had an experience they felt the TRC should know about also reported significantly higher distress (β = 0.265, *p* < 0.01). A significant association was also found between attending the TRC and higher levels of *anger* (β = 0.132, *p* < 0.001).
Kaminer et al. (2001), South Africa		No significant association was found between TRC exposure and PTSD (Χ²=3.62, *p* = 0.16)	No significant association was found between TRC exposure and depression (Χ²=1.63, *p* = 0.44)	No significant association was found between TRC exposure and anxiety (Χ²=0.54, *p* = 0.28)	
Simancas-Fernandez et al. (2022), Colombia	**Colombian Peace Accord reparations**				*Quality of life* or *satisfaction with mental health*: No significant effect by type of government aid received on overall quality of life, physical health), mental health, or environmental factors.
Qouta et al. (1995), Israel-Palestine	**Israel and Palestine peace treaty**				*Neuroticism* decreased significantly between the first (M = 7.40, SD = 2.87) and the second (M = 5.76, SD = 3.09) assessments, t(63) = 4.43, *p* = 0.0001. *Self-esteem* did not significantly change before and after the peace treaty (t(63) = 0.70, p = NS).
Best et al. (2011), Liberia	**Media intervention for Truth and Reconciliation Commission in Liberia**				*Self-efficacy* significantly increased in the treatment group between pre- and post-exposure GSE scores (t [37] = 2.96, *p* = 0.005), with a mean increase of 1.2 points (M = 1.2, SD = 0.39). The control group showed no significant change (t [42] = 0.53, *p* = 0.6). A significant interaction effect between pre- and post-GSE scores across groups was found (F = 3.9, *p* = 0.049).

In bold p values that are statistically significant; p = NS refers to p value = non-significant, no specific p value was reported by the author

One individual-level intervention was identified. Adhikari et al. [42] assessed a rehabilitation programme for former child soldiers in Nepal that offered them four pathways: formal education, micro-enterprise financing, vocational training, and health sector training, finding significant reductions in anxiety and PTSD, but no change in depression. Outcomes varied by rehabilitation type: boys in vocational or health-sector training had increased PTSD, and those in health training also showed more depression. No significant changes were observed among girls. As training was not randomly assigned, results may reflect selection bias or intervention effects.

Two family-level interventions were found. In Colombia, Leon-Jurado and Maldonado [51] studied the intervention “Transformando Mi Futuro” (Transforming My Future), which aimed to provide victims of armed conflict with tools to optimise the use of their monetary reparations. Positive effects were reported on well-being, self-perception, and self-esteem. No controls were included. Glass et al. [43] evaluated the Pigs for Peace intervention, a hybrid microcredit and asset transfer programme in rural Democratic Republic of Congo. Compared to controls, participants showed greater improvements in anxiety and PTSD symptoms, but not depression. The authors suggest benefits stemmed from stress reduction and alternatives to cash loans.

Seven community-level interventions were identified. Five focused on community truth-telling (four in Rwanda, one in Sierra Leone) and two on intergroup contact. The four Rwandan studies examined the Gacaca courts, a community-based transitional justice mechanism established after the 1994 genocide in which accused individuals explained their actions before community-elected judges, survivors, and community members to promote truth and reconciliation. Kanyangara et al. [44] assessed the Gacaca process’s impact on survivors and perpetrators, 45 days before and after trials. All negative emotions except anger increased post-Gacaca, especially among survivors, who reported significantly more fear. Perpetrators showed decreased anger and increased guilt, while survivors showed no changes in these emotions. The authors suggest survivors re-experienced fear, while perpetrators felt heightened guilt. Rimé et al. [52] also examined Gacaca’s emotional and PTSD effects and included a control group. The results mirrored those of Kanyangara et al. [44]: both groups (survivors and perpetrators) experienced more fear, sadness, and anxiety after participation. PTSD symptoms remained unchanged in controls but rose significantly among survivors after the trials.

Brounéus [39] examined the impact of witnessing Gacaca trials on PTSD and depression among survivors, neighbours, and judges in Rwanda. Survivors had greater trauma exposure and higher rates of PTSD and depression than other groups. Across all subgroups, survivors who witnessed Gacaca proceedings reported significantly higher levels of PTSD and depression than those who did not. Overall, individuals who witnessed Gacaca proceedings reported elevated levels of depression and PTSD, even after adjusting for trauma exposure and subgroup membership. Of the four Rwandan studies, only Caparos et al. [45] reported predominantly positive results. This cross-sectional study, conducted 2–4 years after the final Gacaca trials, included survivors and neighbours, grouped by level of participation: non-attendees, attendees without testifying, and attendees who testified. Both survivors and neighbours who attended had lower PTSD symptoms than those who did not. Among survivors, those who attended without testifying reported fewer PTSD symptoms than those who testified. Attendance was also linked to lower depression levels. The authors concluded that Gacaca participation was beneficial only when survivors did not testify.

The second community truth-telling intervention was studied by Cilliers et al. [40], who evaluated *Fambul Tok*, a Sierra Leonean NGO-led reconciliation programme in which victims and perpetrators gathered in community ceremonies to share testimonies, seek forgiveness, and engage in traditional healing rituals. Using a randomized design, the authors assessed the impact of the reconciliation process on trauma, anxiety, and depression, finding negative effects on all three outcomes. These effects persisted for up to 31 months, suggesting that war memories triggered by reconciliation are enduring. Furthermore, the authors found that non-victims can experience a deterioration in mental health as a result of participating in a reconciliation process, likely due to exposure to others’ traumatic experiences.

The two intergroup initiatives were evaluated by Kang et al. [53] and Weder et al. [49]. Kang et al. [53] evaluated *Cows for Peace*, a Rwandan peacebuilding programme promoting reconciliation between genocide survivors and perpetrators. Workshop participation reduced traumatic symptoms in survivors but not perpetrators; while participation in cell groups was associated with a decrease in traumatic symptoms among genocidaires, but not survivors. After a third activity involving both cell groups and cow raising, perpetrators showed decreased PTSD symptoms, while survivors had no change. Overall, both groups reported reductions in traumatic symptoms.

Weder et al. [49] studied the Parents Circle Families Forum (PCFF), a joint Israeli-Palestinian peacebuilding initiative of bereaved families who lost a first-degree relative to the conflict, aiming to transform anger and despair into empathy and hope through a dial-in phone service, a school speaker programme, and international community talks. Among 21 participants (13 PCFF members, 8 non-members), PCFF members showed cognitive, emotional and behavioural symptoms, though differences were not statistically significant.

Five national-level interventions were identified: two evaluated the South African Truth and Reconciliation Commission (TRC), one assessed a media intervention supporting Liberia’s TRC, and two examined the effects of peace treaties or reparations in Palestine and Colombia. The South African TRC aimed to document human rights violations during apartheid, allowing survivors to testify through private statements or public hearings. Perpetrators could receive amnesty in exchange for full disclosure. Stein et al. [46] conducted a cross-sectional study 6–8 years after the TRC using nationally representative data. They assessed distress and anger, finding that participation in TRC events was significantly associated with higher levels of both. Individuals who shared their experiences with the TRC reported higher levels of distress.

Kaminer et al. [41] also conducted a cross-sectional study, 2–3 years after TRC participation, examining the effects of different forms of exposure (testifying, closed statements, or no participation) on PTSD, depression, and anxiety. No significant associations were found between TRC exposure and any of these mental health outcomes. Best et al. [50] examined the role of media in truth-telling and intra-psychic healing in Liberia. The study’s aim was to assess how digital media promote truth-telling and enhance self-efficacy, their perceived ability to effectively cope with stress. Although a control group was included, group allocation was non-random. Results showed that participants exposed to the media-rich intervention had greater increases in self-efficacy than controls. However, those more affected by the war showed smaller gains. The authors suggest that digital media can support intra-psychic healing.

In terms of peace treaties, Quota et al. [48] examined the impact of the 1993 Israel–Palestine peace treaty on the psychological well-being of Palestinian children. Using a pre-post design in randomly selected schools in Gaza refugee camps and urban areas, they assessed neuroticism and self-esteem. Results showed a significant reduction in neuroticism post-treaty, with no change in self-esteem. Children who participated in treaty festivities had lower neuroticism and higher self-esteem than non-participants. Greater trauma exposure was linked to higher neuroticism and lower self-esteem, but only among those who did not participate in the festivities.

The second study on peace treaties focused on Colombia’s internal conflict, which lasted over 50 years and affected more than 9.9 million victims [54]. Simancas-Fernandez et al. [47] studied 1,138 registered victims who received reparations included through the peace agreement with the FARC-EP guerrilla group. Quality of life was assessed using the WHOQOL-BREF [55] which includes a psychological health domain covering positive feelings, spirituality, cognition, self-image, and negative emotions. Results showed that reparations did not significantly improve overall quality of life or mental health satisfaction.

### Quality of included studies

Risk of bias was assessed using the ROBINS-I and RoB 2 tools for cohort, pre-post designs, and randomized trials. Of the nine studies using these designs, most (*n* = 7) [42, 44, 48, 50, 51, 53] had a serious risk of bias, mainly due to confounding and sampling bias. One study [43] showed concerns related to recall bias, interviewer bias, and potential contamination. Only one study, an RCT, demonstrated a low risk of bias [40]. The remaining six studies were evaluated using the JBI Critical Appraisal Checklist for Analytical Cross-Sectional Studies. While all were included, important limitations were identified, including selection bias [46], sampling bias (non-random samples or samples not representative of the population) [41, 45], recall bias [39, 46], lack of information or control on confounders [39, 46], and incomplete or missing information on the validity and reliability of the instruments [45].

## Discussion

This systematic review provides an overall mixed picture of the mental health impacts of peace and conflict resolution interventions. Some studies report positive associations between psychological well-being and rehabilitation programmes, reparations support, financial aid, and reconciliation media. In contrast, several truth-telling interventions were linked to increased psychological distress. Findings on peace treaties were mixed, with some studies showing benefits and others showing no effect. Given the high level of heterogeneity, the generally low quality of the studies and the fact that the majority of the included studies carried a serious risk of bias, these findings should be interpreted as preliminary associations rather than causal effects. Nevertheless, to our knowledge, this is the first systematic review to synthesise quantitative evidence on the mental health impacts of peace and conflict resolution interventions. The patterns identified provide an initial evidence base to inform the design of psychologically sensitive peacebuilding initiatives and guide future research.

A key finding of this review is that truth-telling interventions, such as Rwanda’s Gacaca courts and South Africa’s TRC, were associated with higher levels of psychological distress, particularly among victims (also referred to as survivors) [39, 44, 52] and among those giving testimony [46]. Although these processes are intended to promote reconciliation, they may unintentionally retraumatise participants. Several factors may explain this. First, they may reopen psychological wounds and reactivate sensory-based memories [56–58], heightening anxiety and fear. When trauma exposure is intense and brief, there may be insufficient time for habituation or relearning [59–61]. However, some studies suggest that, given adequate time for memory reconsolidation [62], such reactivation may support psychological closure [45] and provide relief [57].

Importantly, even among those who do participate in this type of intervention, the level of engagement does not carry equal psychological risk, and specific design features may drive differential outcomes. A key distinction emerging from the included studies is between active public testimony and passive participation. Caparos et al. [45] found that survivors who attended the Gacaca but did not testify reported fewer PTSD and depressive symptoms than those who testified. This suggests that the presence alone may carry fewer psychological costs than direct public disclosure. This is further supported by Stein et al. [46] who found that distress was specifically elevated among those who had an experience they felt the TRC should know about, and by Best et al. [50], who found that more indirect, media-based engagement with truth-telling content in Liberia was associated with improved self-efficacy, raising the possibility that mediated forms of participation may be less psychologically harmful than direct public testimony. Beyond the mode of participation, the degree to which involvement is voluntary could also moderate outcomes, given that perceived control over trauma exposure is a well-established buffer against distress [58].

A further distinction worth noting is between the experiences of survivors and perpetrators, who appear to respond to truth-telling processes in different ways. While survivors predominantly exhibited heightened fear, depression and trauma-related symptoms, perpetrators experienced increased guilt alongside reduced anger [39, 44, 45, 52]. Several factors may help explain why survivors appear particularly vulnerable. Survivors are more likely to re-engage directly with traumatic memories during testimony, potentially intensifying emotional reactivation. Beyond the testimony itself, as hypothesised by Brounéus [39], the insecurity experienced by victims as a result of witnessing truth commissions may also contribute to increased distress. Backer [63] found that some individuals who testified at South Africa’s TRC were stigmatised and abandoned by their communities and perceived as traitors. They also received threats before, during, and after their testimony, all of which may have intensified their negative responses. Finally, continued exposure to violence may further explain the limited observed impact of the TRC on psychiatric outcomes. In many contexts, violence persists long after the conflict ends, potentially undermining the effectiveness of these interventions [41].

These findings suggest that truth-telling alone may be insufficient for promoting psychological well-being and that the design of these interventions, particularly the degree of public exposure, participant role, and voluntariness of participation, may be as important for mental health outcomes as the truth-telling process itself. While reconciliation can contribute to societal healing, it often comes at a significant cost to individual recovery [40]. However, when reconciliation processes are embedded within structured therapeutic frameworks, their psychological impact can differ substantially. Kang et al. [53] found that contact between survivors and perpetrators, when combined with CBT, self-help, and cooperative activities, was associated with reductions in trauma symptoms over time. This suggests that peace initiatives incorporating structured psychological support could mitigate the potential distress associated with intergroup confrontation. Such findings are consistent with emerging evidence supporting the integration of cognitive-behavioural strategies with sustained social support in trauma-focused interventions [64].

Interventions without truth-telling components generally showed more positive associations with mental health outcomes. Although difficult to categorise due to high heterogeneity, interventions involving financial elements [43, 51, 53] tended to show positive associations. This aligns with prior evidence from non-conflict settings indicating that cash transfer programmes can improve mental health outcomes [65]. In conflict settings, economic support may improve mental health by alleviating financial stress and promoting economic stability [66]. Furthermore, financial education can positively impact mental health by influencing how individuals process information and make decisions, thereby shaping their perceptions of life opportunities and choices concerning savings, credit, employment, and consumption [51].

The findings of this review have important implications for policymakers and practitioners involved in designing and implementing peace interventions in post-conflict settings. Both the UNDP [29] and the IASC [30] have recently recognised that peace cannot be sustained if the psychological impacts of conflict remain unaddressed, and that programmes combining MHPSS and peacebuilding are likely to achieve greater positive effect than either field working in isolation. Our findings support and extend this policy consensus: although mental health considerations are rarely explicitly integrated into the design of political post-conflict initiatives, the evidence reviewed here suggests that intervention components such as voluntariness, security conditions, and access to psychosocial support may significantly benefit psychological outcomes.

In line with the ‘Do No Harm’ principle highlighted by the IASC [30], incorporating such safeguards into peace processes from the outset could help mitigate unintended psychological harm, particularly in truth-telling and transitional justice contexts. Similarly, the positive associations observed with economic and rehabilitation interventions suggest that restoring material security and agency is a valuable pathway to psychological recovery, and that these components could be more deliberately incorporated into broader peacebuilding programmes.

The systematic review identified several limitations in the included studies. Most relied on cross-sectional designs, small sample sizes or short follow-up periods, limiting the ability to assess long-term effects. Many used non-probability sampling methods, raising concerns about external validity. Another limitation was confounding, as many studies did not control for factors influencing mental health. Small sample sizes and non-adapted instruments further weakened the robustness and reliability of findings.

The studies also have limitations related to data collection. First, in some cases, the measures were administered many years after the intervention, which could lead to inaccurate recall and conflation of the intervention’s impact with that of other traumatic events that occurred in the meantime. Second, some studies only had one follow-up, conducted very close to the intervention, capturing only short- to medium-term effects. Third, the lack of baseline psychological measures prior to the events complicates the interpretation of observational studies. Finally, studies also face difficulties in measuring the impact of interventions. Depending on the perceived source and purpose of the survey, participants may underreport or overreport symptoms [39].

This systematic review also has several limitations. First, the concept of peace is broad and is interpreted differently across studies and within the field. Similarly, there is no agreed set of terms or definitions in conflict resolution [34] and practitioners involved in peacebuilding activities may not use that label in their work. Only peer-reviewed quantitative studies were included, excluding grey literature and qualitative research that could have provided valuable insights into mechanisms, implementation, and heterogeneous experiences across participant subgroups. The considerable heterogeneity in methods, intervention components, measures, populations, and definitions— alongside the high risk of bias in many studies— made it difficult to compare interventions, prevented meta-analysis, and limited our ability to assess the consistency of effects. Furthermore, the socio-ecological framework used to organise the synthesis categorises interventions by the level at which they operate but does not capture the substantial differences in mechanisms. Finally, while the review aimed to address a wide range of mental health and well-being outcomes, it did not examine how psychological mechanisms such as forgiveness, reconciliation, or catharsis might influence or moderate these effects.

Future research should address several important gaps in the existing literature. Long-term follow-up studies are required in order to evaluate the lasting impact of peace interventions on mental health and well-being. Furthermore, future systematic reviews would also benefit from adopting different synthesis frameworks that move beyond descriptive categorisation and better account for the heterogeneous mechanisms through which different types of interventions may affect mental health outcomes. Additionally, more gender-sensitive studies are needed to examine how interventions may affect men, women, and gender-diverse individuals differently. Finally, despite the ethical and logistical challenges involved, more rigorous experimental and quasi-experimental studies are required to strengthen causal inference regarding the mental health effects of peace initiatives in conflict-affected settings.

## Conclusions

This systematic review provides preliminary evidence that peace and conflict resolution interventions have heterogeneous associations with mental health, with outcomes varying substantially by intervention type. Economic and rehabilitation interventions were more consistently associated with positive psychological well-being, whereas truth-telling processes were more frequently associated with increased distress, particularly among survivors and those who testified publicly. These findings highlight that peace interventions are not inherently beneficial for mental health, and that the psychological consequences — both intended and unintended — must be considered when designing and evaluating them. As peacebuilding efforts continue to expand in post-conflict settings worldwide, it is essential that mental health considerations are integrated into their planning to ensure that the pursuit of political reconciliation does not come at the cost of individual psychological recovery.

## Supplementary Information

Supplementary material 1.

## Data Availability

All data relevant to the study are included in the article or uploaded as supplementary information.
