# Supplementary material for: The impacts of peace and conflict resolution interventions on mental health and psychological well-being: a systematic review
Source: Confl Health. 2026 May 31;20:69. doi: 10.1186/s13031-026-00804-2 (PMC13436311; doi:10.1186/s13031-026-00804-2)
Supplement: Supplementary file 1 [file 13031_2026_804_MOESM1_ESM.docx]

**Appendices**

**Appendix A. Search strategies**

i. **General search strategy – 9 April 2026**

(Armed conflict* OR War* OR Post-war* OR Post-conflict* OR Conflict* OR Genocide* OR Forced displacement* OR Civil conflict*) AND (Peace* OR Post-conflict process* OR Reconciliation OR Restoration OR Reparation OR Reintegration OR Forgiveness OR Trust OR “Violence prevention” OR “Social reconstruction” OR Truth* OR “Conflict resolution” OR “Transformative justice” OR “Transitional justice” OR “Restorative justice” OR Dialogue process* OR Retributive justice”) AND (“Mental health” OR Mental disorder* OR Psychiatric disorder* OR Psychiatric diagnos* OR Psychiatric symptom* OR Depression OR Depressive* OR Anxiet* OR Anxious* OR Panic* OR Posttraumatic stress disorder* OR PTSD OR Well-being OR Self-esteem OR Self-confidence OR Self-efficacy OR Resilience OR Insomnia* OR Happiness) AND (Iran or Philippines or India or Pakistan or Myanmar or Burma or Yemen or Guatemala or Israel or Palestine or Iraq or Lebanon or Laos or "DR Congo" or Ethiopia or Nepal or China or Venezuela or Burundi or Chad or Colombia or Indonesia or Peru or Nigeria or Syria or Guinea or Sudan or "Sri Lanka" or Uganda or "United Kingdom" or "El Salvador" or Bangladesh or Eritrea or Mozambique or Afghanistan or Nicaragua or Somalia or Liberia or Spain or Tunisia or Cameroon or Turkey or Togo or "Burkina Faso" or "Papua New Guinea" or Kuwait or Mali or Niger or Rwanda or Senegal or Russia or "Soviet Union" or "Trinidad and Tobago" or Djibouti or Georgia or Haiti or "Sierra Leone" or Serbia or Yugoslavia or Algeria or Angola or Azerbaijan or "Bosnia-Herzegovina" or Croatia or Egypt or Moldova or Tajikistan or Mexico or Ecuador or Comoros or Congo or Guinea-Bissau or Lesotho or Uzbekistan or "Central African Republic" or "North Macedonia" or "United States" or "Ivory Coast" or Australia or Thailand or Mauritania or "South Sudan" or Libya or Malaysia or Ukraine or Kyrgyzstan or Kenya or Jordan or Tanzania or Benin or Bolivia or Cambodia or Greece or France or Thailand or Albania or Vietnam or Madagascar or Paraguay or "Costa Rica" or Yemen or Taiwan or "North Korea" or "South Korea" or Cuba or Morocco or Argentina or Cyprus or Hungary or Honduras or Oman or Brunei or Gabon or "South Yemen" or "Dominican Republic" or Ghana or "South Africa" or Madagascar or Zimbabwe or Uruguay or Chile or "Saudi Arabia" or Gambia or Grenada or Suriname or Panama or Romania or Hyderabad)

ii. **Table 1. Medline (OVID) search strategies and results – 9 April 2026 (1193 results)**

| 1 | Armed conflict*.mp. [mp=title, book title, abstract, original title, name of substance word, subject heading word, floating sub-heading word, keyword heading word, organism supplementary concept word, protocol supplementary concept word, rare disease supplementary concept word, unique identifier, synonyms, population supplementary concept word, anatomy supplementary concept word] | 4294 |
| --- | --- | --- |
| 2 | War*.mp. [mp=title, book title, abstract, original title, name of substance word, subject heading word, floating sub-heading word, keyword heading word, organism supplementary concept word, protocol supplementary concept word, rare disease supplementary concept word, unique identifier, synonyms, population supplementary concept word, anatomy supplementary concept word] | 725853 |
| 3 | exp *War/ | 6489 |
| 4 | Post-war*.mp. [mp=title, book title, abstract, original title, name of substance word, subject heading word, floating sub-heading word, keyword heading word, organism supplementary concept word, protocol supplementary concept word, rare disease supplementary concept word, unique identifier, synonyms, population supplementary concept word, anatomy supplementary concept word] | 2359 |
| 5 | Post-conflict*.mp. [mp=title, book title, abstract, original title, name of substance word, subject heading word, floating sub-heading word, keyword heading word, organism supplementary concept word, protocol supplementary concept word, rare disease supplementary concept word, unique identifier, synonyms, population supplementary concept word, anatomy supplementary concept word] | 1252 |
| 6 | Conflict*.mp. [mp=title, book title, abstract, original title, name of substance word, subject heading word, floating sub-heading word, keyword heading word, organism supplementary concept word, protocol supplementary concept word, rare disease supplementary concept word, unique identifier, synonyms, population supplementary concept word, anatomy supplementary concept word] | 200151 |
| 7 | Genocide*.mp. [mp=title, book title, abstract, original title, name of substance word, subject heading word, floating sub-heading word, keyword heading word, organism supplementary concept word, protocol supplementary concept word, rare disease supplementary concept word, unique identifier, synonyms, population supplementary concept word, anatomy supplementary concept word] | 976 |
| 8 | exp *Genocide/ | 1009 |
| 9 | Forced displacement*.mp. [mp=title, book title, abstract, original title, name of substance word, subject heading word, floating sub-heading word, keyword heading word, organism supplementary concept word, protocol supplementary concept word, rare disease supplementary concept word, unique identifier, synonyms, population supplementary concept word, anatomy supplementary concept word] | 494 |
| 10 | Civil conflict*.mp. [mp=title, book title, abstract, original title, name of substance word, subject heading word, floating sub-heading word, keyword heading word, organism supplementary concept word, protocol supplementary concept word, rare disease supplementary concept word, unique identifier, synonyms, population supplementary concept word, anatomy supplementary concept word] | 224 |
| 11 | Peace*.mp. [mp=title, book title, abstract, original title, name of substance word, subject heading word, floating sub-heading word, keyword heading word, organism supplementary concept word, protocol supplementary concept word, rare disease supplementary concept word, unique identifier, synonyms, population supplementary concept word, anatomy supplementary concept word] | 10314 |
| 12 | Post-conflict process*.mp. [mp=title, book title, abstract, original title, name of substance word, subject heading word, floating sub-heading word, keyword heading word, organism supplementary concept word, protocol supplementary concept word, rare disease supplementary concept word, unique identifier, synonyms, population supplementary concept word, anatomy supplementary concept word] | 5 |
| 13 | Reconciliation.mp. [mp=title, book title, abstract, original title, name of substance word, subject heading word, floating sub-heading word, keyword heading word, organism supplementary concept word, protocol supplementary concept word, rare disease supplementary concept word, unique identifier, synonyms, population supplementary concept word, anatomy supplementary concept word] | 7765 |
| 14 | Restoration.mp. [mp=title, book title, abstract, original title, name of substance word, subject heading word, floating sub-heading word, keyword heading word, organism supplementary concept word, protocol supplementary concept word, rare disease supplementary concept word, unique identifier, synonyms, population supplementary concept word, anatomy supplementary concept word] | 180279 |
| 15 | Reparation.mp. [mp=title, book title, abstract, original title, name of substance word, subject heading word, floating sub-heading word, keyword heading word, organism supplementary concept word, protocol supplementary concept word, rare disease supplementary concept word, unique identifier, synonyms, population supplementary concept word, anatomy supplementary concept word] | 3553 |
| 16 | Reintegration.mp. [mp=title, book title, abstract, original title, name of substance word, subject heading word, floating sub-heading word, keyword heading word, organism supplementary concept word, protocol supplementary concept word, rare disease supplementary concept word, unique identifier, synonyms, population supplementary concept word, anatomy supplementary concept word] | 5096 |
| 17 | Forgiveness.mp. [mp=title, book title, abstract, original title, name of substance word, subject heading word, floating sub-heading word, keyword heading word, organism supplementary concept word, protocol supplementary concept word, rare disease supplementary concept word, unique identifier, synonyms, population supplementary concept word, anatomy supplementary concept word] | 1802 |
| 18 | exp *Forgiveness/ | 527 |
| 19 | Trust.mp. [mp=title, book title, abstract, original title, name of substance word, subject heading word, floating sub-heading word, keyword heading word, organism supplementary concept word, protocol supplementary concept word, rare disease supplementary concept word, unique identifier, synonyms, population supplementary concept word, anatomy supplementary concept word] | 73558 |
| 20 | exp *Trust/ | 7067 |
| 21 | "Violence prevention".mp. [mp=title, book title, abstract, original title, name of substance word, subject heading word, floating sub-heading word, keyword heading word, organism supplementary concept word, protocol supplementary concept word, rare disease supplementary concept word, unique identifier, synonyms, population supplementary concept word, anatomy supplementary concept word] | 3518 |
| 22 | "Social reconstruction".mp. [mp=title, book title, abstract, original title, name of substance word, subject heading word, floating sub-heading word, keyword heading word, organism supplementary concept word, protocol supplementary concept word, rare disease supplementary concept word, unique identifier, synonyms, population supplementary concept word, anatomy supplementary concept word] | 52 |
| 23 | Truth*.mp. [mp=title, book title, abstract, original title, name of substance word, subject heading word, floating sub-heading word, keyword heading word, organism supplementary concept word, protocol supplementary concept word, rare disease supplementary concept word, unique identifier, synonyms, population supplementary concept word, anatomy supplementary concept word] | 42364 |
| 24 | "Conflict resolution".mp. [mp=title, book title, abstract, original title, name of substance word, subject heading word, floating sub-heading word, keyword heading word, organism supplementary concept word, protocol supplementary concept word, rare disease supplementary concept word, unique identifier, synonyms, population supplementary concept word, anatomy supplementary concept word] | 2914 |
| 25 | exp *Conflict Resolution/ | 3792 |
| 26 | "Transformative justice".mp. [mp=title, book title, abstract, original title, name of substance word, subject heading word, floating sub-heading word, keyword heading word, organism supplementary concept word, protocol supplementary concept word, rare disease supplementary concept word, unique identifier, synonyms, population supplementary concept word, anatomy supplementary concept word] | 22 |
| 27 | "Transitional justice".mp. [mp=title, book title, abstract, original title, name of substance word, subject heading word, floating sub-heading word, keyword heading word, organism supplementary concept word, protocol supplementary concept word, rare disease supplementary concept word, unique identifier, synonyms, population supplementary concept word, anatomy supplementary concept word] | 55 |
| 28 | "Restorative justice".mp. [mp=title, book title, abstract, original title, name of substance word, subject heading word, floating sub-heading word, keyword heading word, organism supplementary concept word, protocol supplementary concept word, rare disease supplementary concept word, unique identifier, synonyms, population supplementary concept word, anatomy supplementary concept word] | 288 |
| 29 | Dialogue process*.mp. [mp=title, book title, abstract, original title, name of substance word, subject heading word, floating sub-heading word, keyword heading word, organism supplementary concept word, protocol supplementary concept word, rare disease supplementary concept word, unique identifier, synonyms, population supplementary concept word, anatomy supplementary concept word] | 58 |
| 30 | "Retributive justice".mp. [mp=title, book title, abstract, original title, name of substance word, subject heading word, floating sub-heading word, keyword heading word, organism supplementary concept word, protocol supplementary concept word, rare disease supplementary concept word, unique identifier, synonyms, population supplementary concept word, anatomy supplementary concept word] | 51 |
| 31 | Mental health.mp. [mp=title, book title, abstract, original title, name of substance word, subject heading word, floating sub-heading word, keyword heading word, organism supplementary concept word, protocol supplementary concept word, rare disease supplementary concept word, unique identifier, synonyms, population supplementary concept word, anatomy supplementary concept word] | 354349 |
| 32 | exp *Mental Health/ | 41667 |
| 33 | Mental disorder*.mp. [mp=title, book title, abstract, original title, name of substance word, subject heading word, floating sub-heading word, keyword heading word, organism supplementary concept word, protocol supplementary concept word, rare disease supplementary concept word, unique identifier, synonyms, population supplementary concept word, anatomy supplementary concept word] | 246609 |
| 34 | exp *Mental Disorders/ | 1359161 |
| 35 | Psychiatric disorder*.mp. [mp=title, book title, abstract, original title, name of substance word, subject heading word, floating sub-heading word, keyword heading word, organism supplementary concept word, protocol supplementary concept word, rare disease supplementary concept word, unique identifier, synonyms, population supplementary concept word, anatomy supplementary concept word] | 73905 |
| 36 | Psychiatric diagnos*.mp. [mp=title, book title, abstract, original title, name of substance word, subject heading word, floating sub-heading word, keyword heading word, organism supplementary concept word, protocol supplementary concept word, rare disease supplementary concept word, unique identifier, synonyms, population supplementary concept word, anatomy supplementary concept word] | 13431 |
| 37 | Psychiatric symptom*.mp. [mp=title, book title, abstract, original title, name of substance word, subject heading word, floating sub-heading word, keyword heading word, organism supplementary concept word, protocol supplementary concept word, rare disease supplementary concept word, unique identifier, synonyms, population supplementary concept word, anatomy supplementary concept word] | 17504 |
| 38 | Depression*.mp. [mp=title, book title, abstract, original title, name of substance word, subject heading word, floating sub-heading word, keyword heading word, organism supplementary concept word, protocol supplementary concept word, rare disease supplementary concept word, unique identifier, synonyms, population supplementary concept word, anatomy supplementary concept word] | 589116 |
| 39 | Depressive*.mp. [mp=title, book title, abstract, original title, name of substance word, subject heading word, floating sub-heading word, keyword heading word, organism supplementary concept word, protocol supplementary concept word, rare disease supplementary concept word, unique identifier, synonyms, population supplementary concept word, anatomy supplementary concept word] | 251761 |
| 40 | exp *Affective Disorders/ or exp *"Emotional and Behavioral Disorders"/ | 147286 |
| 41 | Anxiet*.mp. [mp=title, book title, abstract, original title, name of substance word, subject heading word, floating sub-heading word, keyword heading word, organism supplementary concept word, protocol supplementary concept word, rare disease supplementary concept word, unique identifier, synonyms, population supplementary concept word, anatomy supplementary concept word] | 387577 |
| 42 | exp *Anxiety Disorders/ or exp *Anxiety/ | 125608 |
| 43 | Anxious*.mp. [mp=title, book title, abstract, original title, name of substance word, subject heading word, floating sub-heading word, keyword heading word, organism supplementary concept word, protocol supplementary concept word, rare disease supplementary concept word, unique identifier, synonyms, population supplementary concept word, anatomy supplementary concept word] | 24185 |
| 44 | Panic*.mp. [mp=title, book title, abstract, original title, name of substance word, subject heading word, floating sub-heading word, keyword heading word, organism supplementary concept word, protocol supplementary concept word, rare disease supplementary concept word, unique identifier, synonyms, population supplementary concept word, anatomy supplementary concept word] | 29008 |
| 45 | exp *Panic Attack/ or exp *Panic Disorder/ | 5790 |
| 46 | Posttraumatic stress disorder*.mp. [mp=title, book title, abstract, original title, name of substance word, subject heading word, floating sub-heading word, keyword heading word, organism supplementary concept word, protocol supplementary concept word, rare disease supplementary concept word, unique identifier, synonyms, population supplementary concept word, anatomy supplementary concept word] | 28171 |
| 47 | PTSD.mp. [mp=title, book title, abstract, original title, name of substance word, subject heading word, floating sub-heading word, keyword heading word, organism supplementary concept word, protocol supplementary concept word, rare disease supplementary concept word, unique identifier, synonyms, population supplementary concept word, anatomy supplementary concept word] | 41955 |
| 48 | Well-being.mp. [mp=title, book title, abstract, original title, name of substance word, subject heading word, floating sub-heading word, keyword heading word, organism supplementary concept word, protocol supplementary concept word, rare disease supplementary concept word, unique identifier, synonyms, population supplementary concept word, anatomy supplementary concept word] | 168400 |
| 49 | Self-esteem.mp. [mp=title, book title, abstract, original title, name of substance word, subject heading word, floating sub-heading word, keyword heading word, organism supplementary concept word, protocol supplementary concept word, rare disease supplementary concept word, unique identifier, synonyms, population supplementary concept word, anatomy supplementary concept word] | 29944 |
| 50 | exp Self-Esteem/ | 0 |
| 51 | Self-confidence.mp. [mp=title, book title, abstract, original title, name of substance word, subject heading word, floating sub-heading word, keyword heading word, organism supplementary concept word, protocol supplementary concept word, rare disease supplementary concept word, unique identifier, synonyms, population supplementary concept word, anatomy supplementary concept word] | 7208 |
| 52 | exp Self-Confidence/ | 0 |
| 53 | Self-efficacy.mp. [mp=title, book title, abstract, original title, name of substance word, subject heading word, floating sub-heading word, keyword heading word, organism supplementary concept word, protocol supplementary concept word, rare disease supplementary concept word, unique identifier, synonyms, population supplementary concept word, anatomy supplementary concept word] | 62834 |
| 54 | exp Self-Efficacy/ | 0 |
| 55 | Resilience.mp. [mp=title, book title, abstract, original title, name of substance word, subject heading word, floating sub-heading word, keyword heading word, organism supplementary concept word, protocol supplementary concept word, rare disease supplementary concept word, unique identifier, synonyms, population supplementary concept word, anatomy supplementary concept word] | 86945 |
| 56 | exp *"Resilience (Psychological)"/ | 0 |
| 57 | Insomnia*.mp. [mp=title, book title, abstract, original title, name of substance word, subject heading word, floating sub-heading word, keyword heading word, organism supplementary concept word, protocol supplementary concept word, rare disease supplementary concept word, unique identifier, synonyms, population supplementary concept word, anatomy supplementary concept word] | 38831 |
| 58 | exp *Insomnia/ | 16868 |
| 59 | Happiness.mp. [mp=title, book title, abstract, original title, name of substance word, subject heading word, floating sub-heading word, keyword heading word, organism supplementary concept word, protocol supplementary concept word, rare disease supplementary concept word, unique identifier, synonyms, population supplementary concept word, anatomy supplementary concept word] | 14909 |
| 60 | exp *Happiness/ | 3152 |
| 61 | (Iran or Philippines or India or Pakistan or Myanmar or Burma or Yemen or Guatemala or Israel or Palestine or Iraq or Lebanon or Laos or "DR Congo" or Ethiopia or Nepal or China or Venezuela or Burundi or Chad or Colombia or Indonesia or Peru or Nigeria or Syria or Guinea or Sudan or "Sri Lanka" or Uganda or "United Kingdom" or "El Salvador" or Bangladesh or Eritrea or Mozambique or Afghanistan or Nicaragua or Somalia or Liberia or Spain or Tunisia or Cameroon or Turkey or Togo or "Burkina Faso" or "Papua New Guinea" or Kuwait or Mali or Niger or Rwanda or Senegal or Russia or "Soviet Union" or "Trinidad and Tobago" or Djibouti or Georgia or Haiti or "Sierra Leone" or Serbia or Yugoslavia or Algeria or Angola or Azerbaijan or "Bosnia-Herzegovina" or Croatia or Egypt or Moldova or Tajikistan or Mexico or Ecuador or Comoros or Congo or Guinea-Bissau or Lesotho or Uzbekistan or "Central African Republic" or "North Macedonia" or "United States" or "Ivory Coast" or Australia or Thailand or Mauritania or "South Sudan" or Libya or Malaysia or Ukraine or Kyrgyzstan or Kenya or Jordan or Tanzania or Benin or Bolivia or Cambodia or Greece or France or Thailand or Albania or Vietman or Madagascar or Paraguay or "Costa Rica" or Yemen or Taiwan or "North Korea" or "South Korea" or Cuba or Morocco or Argentina or Cyprus or Hungary or Honduras or Oman or Brunei or Gabon or "South Yemen" or "Dominican Republic" or Ghana or "South Africa" or Madagascar or Zimbabwe or Uruguay or Chile or "Saudi Arabia" or Gambia or Grenada or Suriname or Panama or Romania or Hyderabad).mp. [mp=title, book title, abstract, original title, name of substance word, subject heading word, floating sub-heading word, keyword heading word, organism supplementary concept word, protocol supplementary concept word, rare disease supplementary concept word, unique identifier, synonyms, population supplementary concept word, anatomy supplementary concept word] | 4217389 |
| 62 | 1 or 2 or 3 or 4 or 5 or 6 or 7 or 8 or 9 or 10 | 914279 |
| 63 | 11 or 12 or 13 or 14 or 15 or 16 or 17 or 18 or 19 or 20 or 21 or 22 or 23 or 24 or 25 or 26 or 27 or 28 or 29 or 30 | 331856 |
| 64 | 31 or 32 or 33 or 34 or 35 or 36 or 37 or 38 or 39 or 40 or 41 or 42 or 43 or 44 or 45 or 46 or 47 or 48 or 49 or 50 or 51 or 52 or 53 or 54 or 55 or 56 or 57 or 58 or 59 or 60 | 2433402 |
| 65 | 61 and 62 and 63 and 64 | 1230 |
| 66 | exp animals/ not humans.sh. | 5443316 |
| 67 | 65 not 66 | 1213 |
| 68 | limit 67 to yr="1990 -Current" | 1193 |

iii. **Table 2. Embase (OVID) search strategies and results – 9 April 2026 (75 results)**

| 1 | Armed conflict*.mp. [mp=title, abstract, heading word, drug trade name, original title, device manufacturer, drug manufacturer, device trade name, keyword heading word, floating subheading word, candidate term word] | 2973 |
| --- | --- | --- |
| 2 | War*.mp. [mp=title, abstract, heading word, drug trade name, original title, device manufacturer, drug manufacturer, device trade name, keyword heading word, floating subheading word, candidate term word] | 1467519 |
| 3 | exp *War/ | 20028 |
| 4 | Post-war*.mp. [mp=title, abstract, heading word, drug trade name, original title, device manufacturer, drug manufacturer, device trade name, keyword heading word, floating subheading word, candidate term word] | 3452 |
| 5 | Post-conflict*.mp. [mp=title, abstract, heading word, drug trade name, original title, device manufacturer, drug manufacturer, device trade name, keyword heading word, floating subheading word, candidate term word] | 1279 |
| 6 | Conflict*.mp. [mp=title, abstract, heading word, drug trade name, original title, device manufacturer, drug manufacturer, device trade name, keyword heading word, floating subheading word, candidate term word] | 316741 |
| 7 | exp *Conflict/ | 11583 |
| 8 | Genocide*.mp. [mp=title, abstract, heading word, drug trade name, original title, device manufacturer, drug manufacturer, device trade name, keyword heading word, floating subheading word, candidate term word] | 1322 |
| 9 | exp *Genocide/ | 284 |
| 10 | Forced displacement*.mp. [mp=title, abstract, heading word, drug trade name, original title, device manufacturer, drug manufacturer, device trade name, keyword heading word, floating subheading word, candidate term word] | 500 |
| 11 | exp *Forced migration/ | 164 |
| 12 | Civil conflict*.mp. [mp=title, abstract, heading word, drug trade name, original title, device manufacturer, drug manufacturer, device trade name, keyword heading word, floating subheading word, candidate term word] | 236 |
| 13 | Peace*.mp. [mp=title, abstract, heading word, drug trade name, original title, device manufacturer, drug manufacturer, device trade name, keyword heading word, floating subheading word, candidate term word] | 13872 |
| 14 | Post-conflict process*.mp. [mp=title, abstract, heading word, drug trade name, original title, device manufacturer, drug manufacturer, device trade name, keyword heading word, floating subheading word, candidate term word] | 2 |
| 15 | Reconciliation.mp. [mp=title, abstract, heading word, drug trade name, original title, device manufacturer, drug manufacturer, device trade name, keyword heading word, floating subheading word, candidate term word] | 11887 |
| 16 | Restoration.mp. [mp=title, abstract, heading word, drug trade name, original title, device manufacturer, drug manufacturer, device trade name, keyword heading word, floating subheading word, candidate term word] | 202183 |
| 17 | Reparation.mp. [mp=title, abstract, heading word, drug trade name, original title, device manufacturer, drug manufacturer, device trade name, keyword heading word, floating subheading word, candidate term word] | 4883 |
| 18 | Reintegration.mp. [mp=title, abstract, heading word, drug trade name, original title, device manufacturer, drug manufacturer, device trade name, keyword heading word, floating subheading word, candidate term word] | 7853 |
| 19 | Forgiveness.mp. [mp=title, abstract, heading word, drug trade name, original title, device manufacturer, drug manufacturer, device trade name, keyword heading word, floating subheading word, candidate term word] | 2331 |
| 20 | exp *Forgiveness/ | 770 |
| 21 | Trust.mp. [mp=title, abstract, heading word, drug trade name, original title, device manufacturer, drug manufacturer, device trade name, keyword heading word, floating subheading word, candidate term word] | 113932 |
| 22 | exp *Trust/ | 9794 |
| 23 | "Violence prevention".mp. [mp=title, abstract, heading word, drug trade name, original title, device manufacturer, drug manufacturer, device trade name, keyword heading word, floating subheading word, candidate term word] | 3914 |
| 24 | "Social reconstruction".mp. [mp=title, abstract, heading word, drug trade name, original title, device manufacturer, drug manufacturer, device trade name, keyword heading word, floating subheading word, candidate term word] | 53 |
| 25 | Truth*.mp. [mp=title, abstract, heading word, drug trade name, original title, device manufacturer, drug manufacturer, device trade name, keyword heading word, floating subheading word, candidate term word] | 38886 |
| 26 | exp *Truth/ | 0 |
| 27 | "Conflict resolution".mp. [mp=title, abstract, heading word, drug trade name, original title, device manufacturer, drug manufacturer, device trade name, keyword heading word, floating subheading word, candidate term word] | 3522 |
| 28 | "Transformative justice".mp. [mp=title, abstract, heading word, drug trade name, original title, device manufacturer, drug manufacturer, device trade name, keyword heading word, floating subheading word, candidate term word] | 21 |
| 29 | "Transitional justice".mp. [mp=title, abstract, heading word, drug trade name, original title, device manufacturer, drug manufacturer, device trade name, keyword heading word, floating subheading word, candidate term word] | 49 |
| 30 | "Restorative justice".mp. [mp=title, abstract, heading word, drug trade name, original title, device manufacturer, drug manufacturer, device trade name, keyword heading word, floating subheading word, candidate term word] | 346 |
| 31 | exp *Restorative Justice/ | 51 |
| 32 | Dialogue process*.mp. [mp=title, abstract, heading word, drug trade name, original title, device manufacturer, drug manufacturer, device trade name, keyword heading word, floating subheading word, candidate term word] | 72 |
| 33 | "Retributive justice".mp. [mp=title, abstract, heading word, drug trade name, original title, device manufacturer, drug manufacturer, device trade name, keyword heading word, floating subheading word, candidate term word] | 69 |
| 34 | exp *Retributive justice/ | 4 |
| 35 | Mental health.mp. [mp=title, abstract, heading word, drug trade name, original title, device manufacturer, drug manufacturer, device trade name, keyword heading word, floating subheading word, candidate term word] | 551357 |
| 36 | exp *Mental Health/ | 112905 |
| 37 | Mental disorder*.mp. [mp=title, abstract, heading word, drug trade name, original title, device manufacturer, drug manufacturer, device trade name, keyword heading word, floating subheading word, candidate term word] | 108314 |
| 38 | exp *Mental Disorders/ or exp *Mental disease/ | 2113871 |
| 39 | Psychiatric disorder*.mp. [mp=title, abstract, heading word, drug trade name, original title, device manufacturer, drug manufacturer, device trade name, keyword heading word, floating subheading word, candidate term word] | 93527 |
| 40 | Psychiatric diagnos*.mp. [mp=title, abstract, heading word, drug trade name, original title, device manufacturer, drug manufacturer, device trade name, keyword heading word, floating subheading word, candidate term word] | 35260 |
| 41 | Psychiatric symptom*.mp. [mp=title, abstract, heading word, drug trade name, original title, device manufacturer, drug manufacturer, device trade name, keyword heading word, floating subheading word, candidate term word] | 26949 |
| 42 | Depression*.mp. [mp=title, abstract, heading word, drug trade name, original title, device manufacturer, drug manufacturer, device trade name, keyword heading word, floating subheading word, candidate term word] | 1090735 |
| 43 | exp *Depression/ | 357731 |
| 44 | Depressive*.mp. [mp=title, abstract, heading word, drug trade name, original title, device manufacturer, drug manufacturer, device trade name, keyword heading word, floating subheading word, candidate term word] | 264483 |
| 45 | exp *Affective Disorders/ or exp *"Emotional and Behavioral Disorders"/ | 382251 |
| 46 | Anxiet*.mp. [mp=title, abstract, heading word, drug trade name, original title, device manufacturer, drug manufacturer, device trade name, keyword heading word, floating subheading word, candidate term word] | 671148 |
| 47 | exp *Anxiety Disorders/ or exp *Anxiety/ | 257343 |
| 48 | Anxious*.mp. [mp=title, abstract, heading word, drug trade name, original title, device manufacturer, drug manufacturer, device trade name, keyword heading word, floating subheading word, candidate term word] | 36579 |
| 49 | Panic*.mp. [mp=title, abstract, heading word, drug trade name, original title, device manufacturer, drug manufacturer, device trade name, keyword heading word, floating subheading word, candidate term word] | 48664 |
| 50 | exp *Panic Attack/ or exp *Panic Disorder/ | 11019 |
| 51 | Posttraumatic stress disorder*.mp. [mp=title, abstract, heading word, drug trade name, original title, device manufacturer, drug manufacturer, device trade name, keyword heading word, floating subheading word, candidate term word] | 106878 |
| 52 | exp *Posttraumatic Stress Disorder/ | 50004 |
| 53 | PTSD.mp. [mp=title, abstract, heading word, drug trade name, original title, device manufacturer, drug manufacturer, device trade name, keyword heading word, floating subheading word, candidate term word] | 57346 |
| 54 | Well-being.mp. [mp=title, abstract, heading word, drug trade name, original title, device manufacturer, drug manufacturer, device trade name, keyword heading word, floating subheading word, candidate term word] | 245567 |
| 55 | exp *Well Being/ | 35439 |
| 56 | Self-esteem.mp. [mp=title, abstract, heading word, drug trade name, original title, device manufacturer, drug manufacturer, device trade name, keyword heading word, floating subheading word, candidate term word] | 49903 |
| 57 | exp Self-Esteem/ | 33141 |
| 58 | Self-confidence.mp. [mp=title, abstract, heading word, drug trade name, original title, device manufacturer, drug manufacturer, device trade name, keyword heading word, floating subheading word, candidate term word] | 10501 |
| 59 | Self-efficacy.mp. [mp=title, abstract, heading word, drug trade name, original title, device manufacturer, drug manufacturer, device trade name, keyword heading word, floating subheading word, candidate term word] | 67569 |
| 60 | exp Self-Efficacy/ | 324150 |
| 61 | Resilience.mp. [mp=title, abstract, heading word, drug trade name, original title, device manufacturer, drug manufacturer, device trade name, keyword heading word, floating subheading word, candidate term word] | 99091 |
| 62 | exp *"Resilience (Psychological)"/ | 10122 |
| 63 | Insomnia*.mp. [mp=title, abstract, heading word, drug trade name, original title, device manufacturer, drug manufacturer, device trade name, keyword heading word, floating subheading word, candidate term word] | 122094 |
| 64 | exp *Insomnia/ | 28484 |
| 65 | Happiness.mp. [mp=title, abstract, heading word, drug trade name, original title, device manufacturer, drug manufacturer, device trade name, keyword heading word, floating subheading word, candidate term word] | 20146 |
| 66 | exp *Happiness/ | 3773 |
| 67 | (Iran or Philippines or India or Pakistan or Myanmar or Burma or Yemen or Guatemala or Israel or Palestine or Iraq or Lebanon or Laos or "DR Congo" or Ethiopia or Nepal or China or Venezuela or Burundi or Chad or Colombia or Indonesia or Peru or Nigeria or Syria or Guinea or Sudan or "Sri Lanka" or Uganda or "United Kingdom" or "El Salvador" or Bangladesh or Eritrea or Mozambique or Afghanistan or Nicaragua or Somalia or Liberia or Spain or Tunisia or Cameroon or Turkey or Togo or "Burkina Faso" or "Papua New Guinea" or Kuwait or Mali or Niger or Rwanda or Senegal or Russia or "Soviet Union" or "Trinidad and Tobago" or Djibouti or Georgia or Haiti or "Sierra Leone" or Serbia or Yugoslavia or Algeria or Angola or Azerbaijan or "Bosnia-Herzegovina" or Croatia or Egypt or Moldova or Tajikistan or Mexico or Ecuador or Comoros or Congo or Guinea-Bissau or Lesotho or Uzbekistan or "Central African Republic" or "North Macedonia" or "United States" or "Ivory Coast" or Australia or Thailand or Mauritania or "South Sudan" or Libya or Malaysia or Ukraine or Kyrgyzstan or Kenya or Jordan or Tanzania or Benin or Bolivia or Cambodia or Greece or France or Thailand or Albania or Vietman or Madagascar or Paraguay or "Costa Rica" or Yemen or Taiwan or "North Korea" or "South Korea" or Cuba or Morocco or Argentina or Cyprus or Hungary or Honduras or Oman or Brunei or Gabon or "South Yemen" or "Dominican Republic" or Ghana or "South Africa" or Madagascar or Zimbabwe or Uruguay or Chile or "Saudi Arabia" or Gambia or Grenada or Suriname or Panama or Romania or Hyderabad).mp. [mp=title, abstract, heading word, drug trade name, original title, device manufacturer, drug manufacturer, device trade name, keyword heading word, floating subheading word, candidate term word] | 6836996 |
| 68 | 1 or 2 or 3 or 4 or 5 or 6 or 7 or 8 or 9 or 10 or 11 or 12 | 1761649 |
| 69 | 13 or 14 or 15 or 16 or 17 or 18 or 19 or 20 or 21 or 22 or 23 or 24 or 25 or 26 or 27 or 28 or 29 or 30 or 31 or 32 or 33 or 34 | 400500 |
| 70 | 35 or 36 or 37 or 38 or 39 or 40 or 41 or 42 or 43 or 44 or 45 or 46 or 47 or 48 or 49 or 50 or 51 or 52 or 53 or 54 or 55 or 56 or 57 or 58 or 59 or 60 or 61 or 62 or 63 or 64 or 65 or 66 | 3757373 |
| 71 | 67 and 68 and 69 and 70 | 1780 |
| 72 | exp animals/ not humans.sh. | 38364986 |
| 73 | 71 not 72 | 89 |
| 74 | limit 73 to yr="1990 -Current" | 75 |

iv. **Table 3. APA PsychInfo (OVID) search strategies and results – 9 April 2026 (1790 results)**

| 1 | Armed conflict*.mp. [mp=title, abstract, heading word, table of contents, key concepts, original title, tests & measures, mesh word] | 1908 |
| --- | --- | --- |
| 2 | War*.mp. [mp=title, abstract, heading word, table of contents, key concepts, original title, tests & measures, mesh word] | 168353 |
| 3 | exp *War/ | 12807 |
| 4 | Post-war*.mp. [mp=title, abstract, heading word, table of contents, key concepts, original title, tests & measures, mesh word] | 1800 |
| 5 | Post-conflict*.mp. [mp=title, abstract, heading word, table of contents, key concepts, original title, tests & measures, mesh word] | 1393 |
| 6 | Conflict*.mp. [mp=title, abstract, heading word, table of contents, key concepts, original title, tests & measures, mesh word] | 178263 |
| 7 | exp *Conflict/ | 165124 |
| 8 | Genocide*.mp. [mp=title, abstract, heading word, table of contents, key concepts, original title, tests & measures, mesh word] | 2564 |
| 9 | exp *Genocide/ | 2323 |
| 10 | Forced displacement*.mp. [mp=title, abstract, heading word, table of contents, key concepts, original title, tests & measures, mesh word] | 383 |
| 11 | exp *Human Displacement/ | 361 |
| 12 | Civil conflict*.mp. [mp=title, abstract, heading word, table of contents, key concepts, original title, tests & measures, mesh word] | 281 |
| 13 | Peace*.mp. [mp=title, abstract, heading word, table of contents, key concepts, original title, tests & measures, mesh word] | 14941 |
| 14 | exp *Peace/ | 3217 |
| 15 | Post-conflict process*.mp. [mp=title, abstract, heading word, table of contents, key concepts, original title, tests & measures, mesh word] | 6 |
| 16 | Reconciliation.mp. [mp=title, abstract, heading word, table of contents, key concepts, original title, tests & measures, mesh word] | 5885 |
| 17 | Restoration.mp. [mp=title, abstract, heading word, table of contents, key concepts, original title, tests & measures, mesh word] | 9659 |
| 18 | Reparation.mp. [mp=title, abstract, heading word, table of contents, key concepts, original title, tests & measures, mesh word] | 1239 |
| 19 | Reintegration.mp. [mp=title, abstract, heading word, table of contents, key concepts, original title, tests & measures, mesh word] | 5947 |
| 20 | exp *Reintegration/ | 1408 |
| 21 | Forgiveness.mp. [mp=title, abstract, heading word, table of contents, key concepts, original title, tests & measures, mesh word] | 6194 |
| 22 | exp *Forgiveness/ | 3572 |
| 23 | Trust.mp. [mp=title, abstract, heading word, table of contents, key concepts, original title, tests & measures, mesh word] | 58137 |
| 24 | exp *"Trust (Social Behavior)"/ | 15319 |
| 25 | "Violence prevention".mp. [mp=title, abstract, heading word, table of contents, key concepts, original title, tests & measures, mesh word] | 4662 |
| 26 | exp *Violence Prevention/ | 1553 |
| 27 | "Social reconstruction".mp. [mp=title, abstract, heading word, table of contents, key concepts, original title, tests & measures, mesh word] | 164 |
| 28 | Truth*.mp. [mp=title, abstract, heading word, table of contents, key concepts, original title, tests & measures, mesh word] | 28498 |
| 29 | exp *Truth/ | 2666 |
| 30 | "Conflict resolution".mp. [mp=title, abstract, heading word, table of contents, key concepts, original title, tests & measures, mesh word] | 13229 |
| 31 | exp *Conflict Resolution/ | 11635 |
| 32 | "Transformative justice".mp. [mp=title, abstract, heading word, table of contents, key concepts, original title, tests & measures, mesh word] | 86 |
| 33 | "Transitional justice".mp. [mp=title, abstract, heading word, table of contents, key concepts, original title, tests & measures, mesh word] | 344 |
| 34 | "Restorative justice".mp. [mp=title, abstract, heading word, table of contents, key concepts, original title, tests & measures, mesh word] | 1776 |
| 35 | exp *Restorative Justice/ | 629 |
| 36 | Dialogue process*.mp. [mp=title, abstract, heading word, table of contents, key concepts, original title, tests & measures, mesh word] | 122 |
| 37 | "Retributive justice".mp. [mp=title, abstract, heading word, table of contents, key concepts, original title, tests & measures, mesh word] | 271 |
| 38 | Mental health.mp. [mp=title, abstract, heading word, table of contents, key concepts, original title, tests & measures, mesh word] | 346026 |
| 39 | exp *Mental Health/ | 93721 |
| 40 | Mental disorder*.mp. [mp=title, abstract, heading word, table of contents, key concepts, original title, tests & measures, mesh word] | 216616 |
| 41 | exp *Mental Disorders/ | 1051038 |
| 42 | Psychiatric disorder*.mp. [mp=title, abstract, heading word, table of contents, key concepts, original title, tests & measures, mesh word] | 49313 |
| 43 | Psychiatric diagnos*.mp. [mp=title, abstract, heading word, table of contents, key concepts, original title, tests & measures, mesh word] | 14350 |
| 44 | exp *Psychiatric Symptoms/ | 153472 |
| 45 | Psychiatric symptom*.mp. [mp=title, abstract, heading word, table of contents, key concepts, original title, tests & measures, mesh word] | 31786 |
| 46 | Depression*.mp. [mp=title, abstract, heading word, table of contents, key concepts, original title, tests & measures, mesh word] | 441585 |
| 47 | "exp* Depression (Emotion)".mp. [mp=title, abstract, heading word, table of contents, key concepts, original title, tests & measures, mesh word] | 0 |
| 48 | Depressive*.mp. [mp=title, abstract, heading word, table of contents, key concepts, original title, tests & measures, mesh word] | 185711 |
| 49 | exp *Affective Disorders/ or exp *"Emotional and Behavioral Disorders"/ | 182737 |
| 50 | Anxiet*.mp. [mp=title, abstract, heading word, table of contents, key concepts, original title, tests & measures, mesh word] | 337293 |
| 51 | exp *Anxiety Disorders/ or exp *Anxiety/ | 118647 |
| 52 | Anxious*.mp. [mp=title, abstract, heading word, table of contents, key concepts, original title, tests & measures, mesh word] | 28314 |
| 53 | Panic*.mp. [mp=title, abstract, heading word, table of contents, key concepts, original title, tests & measures, mesh word] | 20672 |
| 54 | exp *Panic Attack/ or exp *Panic Disorder/ | 7568 |
| 55 | Posttraumatic stress disorder*.mp. [mp=title, abstract, heading word, table of contents, key concepts, original title, tests & measures, mesh word] | 55062 |
| 56 | exp *Posttraumatic Stress Disorder/ | 40921 |
| 57 | PTSD.mp. [mp=title, abstract, heading word, table of contents, key concepts, original title, tests & measures, mesh word] | 52563 |
| 58 | exp *"Stress and Trauma Related Disorders"/ | 43070 |
| 59 | Well-being.mp. [mp=title, abstract, heading word, table of contents, key concepts, original title, tests & measures, mesh word] | 158183 |
| 60 | exp *Well Being/ | 64465 |
| 61 | Self-esteem.mp. [mp=title, abstract, heading word, table of contents, key concepts, original title, tests & measures, mesh word] | 63497 |
| 62 | exp Self-Esteem/ | 30371 |
| 63 | Self-confidence.mp. [mp=title, abstract, heading word, table of contents, key concepts, original title, tests & measures, mesh word] | 11745 |
| 64 | exp Self-Confidence/ | 5485 |
| 65 | Self-efficacy.mp. [mp=title, abstract, heading word, table of contents, key concepts, original title, tests & measures, mesh word] | 72964 |
| 66 | exp Self-Efficacy/ | 36560 |
| 67 | Resilience.mp. [mp=title, abstract, heading word, table of contents, key concepts, original title, tests & measures, mesh word] | 56849 |
| 68 | exp *"Resilience (Psychological)"/ | 25310 |
| 69 | Insomnia*.mp. [mp=title, abstract, heading word, table of contents, key concepts, original title, tests & measures, mesh word] | 20416 |
| 70 | exp *Insomnia/ | 9011 |
| 71 | Happiness.mp. [mp=title, abstract, heading word, table of contents, key concepts, original title, tests & measures, mesh word] | 25940 |
| 72 | exp *Happiness/ | 8900 |
| 73 | (Iran or Philippines or India or Pakistan or Myanmar or Burma or Yemen or Guatemala or Israel or Palestine or Iraq or Lebanon or Laos or "DR Congo" or Ethiopia or Nepal or China or Venezuela or Burundi or Chad or Colombia or Indonesia or Peru or Nigeria or Syria or Guinea or Sudan or "Sri Lanka" or Uganda or "United Kingdom" or "El Salvador" or Bangladesh or Eritrea or Mozambique or Afghanistan or Nicaragua or Somalia or Liberia or Spain or Tunisia or Cameroon or Turkey or Togo or "Burkina Faso" or "Papua New Guinea" or Kuwait or Mali or Niger or Rwanda or Senegal or Russia or "Soviet Union" or "Trinidad and Tobago" or Djibouti or Georgia or Haiti or "Sierra Leone" or Serbia or Yugoslavia or Algeria or Angola or Azerbaijan or "Bosnia-Herzegovina" or Croatia or Egypt or Moldova or Tajikistan or Mexico or Ecuador or Comoros or Congo or Guinea-Bissau or Lesotho or Uzbekistan or "Central African Republic" or "North Macedonia" or "United States" or "Ivory Coast" or Australia or Thailand or Mauritania or "South Sudan" or Libya or Malaysia or Ukraine or Kyrgyzstan or Kenya or Jordan or Tanzania or Benin or Bolivia or Cambodia or Greece or France or Thailand or Albania or Vietman or Madagascar or Paraguay or "Costa Rica" or Yemen or Taiwan or "North Korea" or "South Korea" or Cuba or Morocco or Argentina or Cyprus or Hungary or Honduras or Oman or Brunei or Gabon or "South Yemen" or "Dominican Republic" or Ghana or "South Africa" or Madagascar or Zimbabwe or Uruguay or Chile or "Saudi Arabia" or Gambia or Grenada or Suriname or Panama or Romania or Hyderabad).mp. [mp=title, abstract, heading word, table of contents, key concepts, original title, tests & measures, mesh word] | 634593 |
| 74 | 1 or 2 or 3 or 4 or 5 or 6 or 7 or 8 or 9 or 10 or 11 or 12 | 445406 |
| 75 | 13 or 14 or 15 or 16 or 17 or 18 or 19 or 20 or 21 or 22 or 23 or 24 or 25 or 26 or 27 or 28 or 29 or 30 or 31 or 32 or 33 or 34 or 35 or 36 or 37 | 145358 |
| 76 | 38 or 39 or 40 or 41 or 42 or 43 or 44 or 45 or 46 or 47 or 48 or 49 or 50 or 51 or 52 or 53 or 54 or 55 or 56 or 57 or 58 or 59 or 60 or 61 or 62 or 63 or 64 or 65 or 66 or 67 or 68 or 69 or 70 or 71 or 72 | 1835897 |
| 77 | 73 and 74 and 75 and 76 | 1842 |
| 78 | exp animals/ not humans.sh. | 398394 |
| 79 | 77 not 78 | 1838 |
| 0 | limit 79 to yr="1990 -Current" | 1790 |

v. **Scopus search strategy and results – 9 April 2026 (2,132 results)**

( TITLE-ABS-KEY ( "Armed conflict" OR "war" OR "post-war" OR "post-conflict" OR conflict* OR genocide* OR "forced displacement" OR "civil conflict" ) AND TITLE-ABS-KEY ( peace* OR "post-conflict process" OR reconciliation OR restoration OR reparation OR reintegration OR forgiveness OR trust OR "violence prevention" OR "social reconstruction" OR truth* OR "conflict resolution" OR "transformative justice" OR "transitional justice" OR "restorative justice" OR "dialogue process" OR "retributive justice" ) AND TITLE-ABS-KEY ( "mental health" OR "mental disorder" OR "psychiatric disorder" OR "psychiatric diagnos*" OR "psychiatric symptom" OR depression OR depressive* OR anxiet* OR anxious* OR panic OR "posttraumatic stress disorder" OR "PTSD" OR "Well-being" OR "self-esteem" OR "self-confidence" OR "self-efficacy" OR resilience OR insomnia* OR happiness ) AND TITLE-ABS-KEY ( iran OR philippines OR india OR pakistan OR myanmar OR burma OR yemen OR guatemala OR israel OR palestine OR iraq OR lebanon OR laos OR "DR Congo" OR ethiopia OR nepal OR china OR venezuela OR burundi OR chad OR colombia OR indonesia OR peru OR nigeria OR syria OR guinea OR sudan OR "Sri Lanka" OR uganda OR "United Kingdom" OR "El Salvador" OR bangladesh OR eritrea OR mozambique OR afghanistan OR nicaragua OR somalia OR liberia OR spain OR tunisia OR cameroon OR turkey OR togo OR "Burkina Faso" OR "Papua New Guinea" OR kuwait OR mali OR niger OR rwanda OR senegal OR russia OR "Soviet Union" OR "Trinidad and Tobago" OR djibouti OR georgia OR haiti OR "Sierra Leone" OR serbia OR yugoslavia OR algeria OR angola OR azerbaijan OR "Bosnia-Herzegovina" OR croatia OR egypt OR moldova OR tajikistan OR mexico OR ecuador OR comoros OR congo OR guinea-bissau OR lesotho OR uzbekistan OR "Central African Republic" OR "North Macedonia" OR "United States" OR "Ivory Coast" OR australia OR thailand OR mauritania OR "South Sudan" OR libya OR malaysia OR ukraine OR kyrgyzstan OR kenya OR jordan OR tanzania OR benin OR bolivia OR cambodia OR greece OR france OR thailand OR albania OR Vietnam OR madagascar OR paraguay OR "Costa Rica" OR yemen OR taiwan OR "North Korea" OR "South Korea" OR cuba OR morocco OR argentina OR cyprus OR hungary OR honduras OR oman OR brunei OR gabon OR "South Yemen" OR "Dominican Republic" OR ghana OR "South Africa" OR madagascar OR zimbabwe OR uruguay OR chile OR "Saudi Arabia" OR gambia OR grenada OR suriname OR panama OR romania OR hyderabad ) ) AND PUBYEAR > 1989 AND ( LIMIT-TO ( DOCTYPE , "ar" ) )

vi. **PTSDpubs search strategy and results - 9 April 2026 (25 results)**

ABSTRACT, TITLE(("ARMED CONFLICT" OR "WAR" OR "GENOCIDE" OR "FORCED DISPLACEMENT" OR "CIVIL CONFLICT")) AND ABSTRACT,TITLE(("PEACE*" OR "POST-CONFLICT PROCESS*" OR RECONCILIATION OR RESTORATION OR REPARATION OR REINTEGRATION OR FORGIVENESS OR TRUST OR "VIOLENCE PREVENTION" OR "SOCIAL RECONSTRUCTION" OR TRUTH* OR "CONFLICT RESOLUTION" OR "TRANSFORMATIVE JUSTICE" OR "TRANSITIONAL JUSTICE" OR "RESTORATIVE JUSTICE" OR ("DIALOGUE PROCESS") OR "RETRIBUTIVE JUSTICE")) AND ABSTRACT,TITLE(("MENTAL HEALTH" OR ("MENTAL DISORDER" OR "MENTAL DISORDERS") OR ("PSYCHIATRIC DISORDER" OR "PSYCHIATRIC DISORDERS") OR ("PSYCHIATRIC DIAGNOSES" OR "PSYCHIATRIC DIAGNOSIS" OR "PSYCHIATRIC DIAGNOSTIC") OR ("PSYCHIATRIC SYMPTOMS") OR DEPRESSION OR DEPRESSIVE* OR ANXIET* OR ANXIOUS* OR PANIC OR "POSTTRAUMATIC STRESS DISORDER" OR "PTSD" OR "WELL-BEING" OR "SELF-ESTEEM" OR "SELF-CONFIDENCE" OR "SELF-EFFICACY" OR "RESILIENCE" OR "INSOMNIA*" OR HAPPINESS)) AND ABSTRACT,TITLE((IRAN OR PHILIPPINES OR INDIA OR PAKISTAN OR MYANMAR OR BURMA OR YEMEN OR GUATEMALA OR ISRAEL OR PALESTINE OR IRAQ OR LEBANON OR LAOS OR "DR CONGO" OR ETHIOPIA OR NEPAL OR CHINA OR VENEZUELA OR BURUNDI OR CHAD OR COLOMBIA OR INDONESIA OR PERU OR NIGERIA OR SYRIA OR GUINEA OR SUDAN OR "SRI LANKA" OR UGANDA OR "UNITED KINGDOM" OR "EL SALVADOR" OR BANGLADESH OR ERITREA OR MOZAMBIQUE OR AFGHANISTAN OR NICARAGUA OR SOMALIA OR LIBERIA OR SPAIN OR TUNISIA OR CAMEROON OR TURKEY OR TOGO OR "BURKINA FASO" OR "PAPUA NEW GUINEA" OR KUWAIT OR MALI OR NIGER OR RWANDA OR SENEGAL OR RUSSIA OR "SOVIET UNION" OR "TRINIDAD AND TOBAGO" OR DJIBOUTI OR GEORGIA OR HAITI OR "SIERRA LEONE" OR SERBIA OR YUGOSLAVIA OR ALGERIA OR ANGOLA OR AZERBAIJAN OR "BOSNIA-HERZEGOVINA" OR CROATIA OR EGYPT OR MOLDOVA OR TAJIKISTAN OR MEXICO OR ECUADOR OR COMOROS OR CONGO OR GUINEA-BISSAU OR LESOTHO OR UZBEKISTAN OR "CENTRAL AFRICAN REPUBLIC" OR "NORTH MACEDONIA" OR "UNITED STATES" OR "IVORY COAST" OR AUSTRALIA OR THAILAND OR MAURITANIA OR "SOUTH SUDAN" OR LIBYA OR MALAYSIA OR UKRAINE OR KYRGYZSTAN OR KENYA OR JORDAN OR TANZANIA OR BENIN OR BOLIVIA OR CAMBODIA OR GREECE OR FRANCE OR THAILAND OR ALBANIA OR VIETNAM OR MADAGASCAR OR PARAGUAY OR "COSTA RICA" OR YEMEN OR TAIWAN OR "NORTH KOREA" OR "SOUTH KOREA" OR CUBA OR MOROCCO OR ARGENTINA OR CYPRUS OR HUNGARY OR HONDURAS OR OMAN OR BRUNEI OR GABON OR "SOUTH YEMEN" OR "DOMINICAN REPUBLIC" OR GHANA OR "SOUTH AFRICA" OR MADAGASCAR OR ZIMBABWE OR URUGUAY OR CHILE OR "SAUDI ARABIA" OR GAMBIA OR GRENADA OR SURINAME OR PANAMA OR ROMANIA OR HYDERABAD))

Limits: After December 31, 1989

Scholarly journals

vii. **LILACS search strategy and results - 9 April 2026 (6 results)**

(Armed conflict* OR War* OR Post-war* OR Post-conflict* OR Conflict* OR Genocide* OR Forced displacement* OR Civil conflict*) AND (Peace* OR Post-conflict process* OR Reconciliation OR Restoration OR Reparation OR Reintegration OR Forgiveness OR Trust OR “Violence prevention” OR “Social reconstruction” OR Truth* OR “Conflict resolution” OR “Transformative justice” OR “Transitional justice” OR “Restorative justice” OR Dialogue process* OR Retributive justice”) AND (“Mental health” OR Mental disorder* OR Psychiatric disorder* OR Psychiatric diagnos* OR Psychiatric symptom* OR Depression OR Depressive* OR Anxiet* OR Anxious* OR Panic* OR Posttraumatic stress disorder* OR PTSD OR Well-being OR Self-esteem OR Self-confidence OR Self-efficacy OR Resilience OR Insomnia* OR Happiness) AND (Iran or Philippines or India or Pakistan or Myanmar or Burma or Yemen or Guatemala or Israel or Palestine or Iraq or Lebanon or Laos or "DR Congo" or Ethiopia or Nepal or China or Venezuela or Burundi or Chad or Colombia or Indonesia or Peru or Nigeria or Syria or Guinea or Sudan or "Sri Lanka" or Uganda or "United Kingdom" or "El Salvador" or Bangladesh or Eritrea or Mozambique or Afghanistan or Nicaragua or Somalia or Liberia or Spain or Tunisia or Cameroon or Turkey or Togo or "Burkina Faso" or "Papua New Guinea" or Kuwait or Mali or Niger or Rwanda or Senegal or Russia or "Soviet Union" or "Trinidad and Tobago" or Djibouti or Georgia or Haiti or "Sierra Leone" or Serbia or Yugoslavia or Algeria or Angola or Azerbaijan or "Bosnia-Herzegovina" or Croatia or Egypt or Moldova or Tajikistan or Mexico or Ecuador or Comoros or Congo or Guinea-Bissau or Lesotho or Uzbekistan or "Central African Republic" or "North Macedonia" or "United States" or "Ivory Coast" or Australia or Thailand or Mauritania or "South Sudan" or Libya or Malaysia or Ukraine or Kyrgyzstan or Kenya or Jordan or Tanzania or Benin or Bolivia or Cambodia or Greece or France or Thailand or Albania or Vietnam or Madagascar or Paraguay or "Costa Rica" or Yemen or Taiwan or "North Korea" or "South Korea" or Cuba or Morocco or Argentina or Cyprus or Hungary or Honduras or Oman or Brunei or Gabon or "South Yemen" or "Dominican Republic" or Ghana or "South Africa" or Madagascar or Zimbabwe or Uruguay or Chile or "Saudi Arabia" or Gambia or Grenada or Suriname or Panama or Romania or Hyderabad)

Filter: articles / & 1990 onwards

viii. **JSTOR search strategy and results - 9 April 2026 (963 results)**

("ARMED CONFLICT" OR "WAR") AND ("PEACE*" OR RECONCILIATION OR TRUTH OR FORGIVENESS OR JUSTICE) AND (“MENTAL HEALTH” OR “WELL-BEING” OR “RESILIENCE” OR "SELF-ESTEEM" OR INSOMNIA OR HAPPINESS) AND (DEPRESSION) AND (ANXIETY) AND (PTSD)

These last three terms are added through the “search within results”

Limited to Journals and 1990 onwards

ix. **Econlit search strategy and results - 9 April 2026 (172 results)**

noft(("ARMED CONFLICT" OR "WAR" OR "GENOCIDE" OR "FORCED DISPLACEMENT" OR "CIVIL CONFLICT")) AND noft(("PEACE*" OR "POST-CONFLICT PROCESS*" OR RECONCILIATION OR RESTORATION OR REPARATION OR REINTEGRATION OR FORGIVENESS OR TRUST OR "VIOLENCE PREVENTION" OR "SOCIAL RECONSTRUCTION" OR TRUTH* OR "CONFLICT RESOLUTION" OR "TRANSFORMATIVE JUSTICE" OR "TRANSITIONAL JUSTICE" OR "RESTORATIVE JUSTICE" OR "DIALOGUE PROCESS*" OR "RETRIBUTIVE JUSTICE")) AND noft(("MENTAL HEALTH" OR "MENTAL DISORDER*" OR "PSYCHIATRIC DISORDER*" OR "PSYCHIATRIC DIAGNOS*" OR "PSYCHIATRIC SYMPTOM*" OR DEPRESSION OR DEPRESSIVE* OR ANXIET* OR ANXIOUS* OR PANIC OR "POSTTRAUMATIC STRESS DISORDER" OR "PTSD" OR "WELL-BEING" OR "SELF-ESTEEM" OR "SELF-CONFIDENCE" OR "SELF-EFFICACY" OR "RESILIENCE" OR "INSOMNIA*" OR HAPPINESS)) AND noft((Iran OR Philippines OR India OR Pakistan OR Myanmar OR Burma OR Yemen OR Guatemala OR Israel OR Palestine OR Iraq OR Lebanon OR Laos OR "DR Congo" OR Ethiopia OR Nepal OR China OR Venezuela OR Burundi OR Chad OR Colombia OR Indonesia OR Peru OR Nigeria OR Syria OR Guinea OR Sudan OR "Sri Lanka" OR Uganda OR "United Kingdom" OR "El Salvador" OR Bangladesh OR Eritrea OR Mozambique OR Afghanistan OR Nicaragua OR Somalia OR Liberia OR Spain OR Tunisia OR Cameroon OR Turkey OR Togo OR "Burkina Faso" OR "Papua New Guinea" OR Kuwait OR Mali OR Niger OR Rwanda OR Senegal OR Russia OR "Soviet Union" OR "Trinidad and Tobago" OR Djibouti OR Georgia OR Haiti OR "Sierra Leone" OR Serbia OR Yugoslavia OR Algeria OR Angola OR Azerbaijan OR "Bosnia-Herzegovina" OR Croatia OR Egypt OR Moldova OR Tajikistan OR Mexico OR Ecuador OR Comoros OR Congo OR Guinea-Bissau OR Lesotho OR Uzbekistan OR "Central African Republic" OR "North Macedonia" OR "United States" OR "Ivory Coast" OR Australia OR Thailand OR Mauritania OR "South Sudan" OR Libya OR Malaysia OR Ukraine OR Kyrgyzstan OR Kenya OR Jordan OR Tanzania OR Benin OR Bolivia OR Cambodia OR Greece OR France OR Thailand OR Albania OR Vietnam OR Madagascar OR Paraguay OR "Costa Rica" OR Yemen OR Taiwan OR "North Korea" OR "South Korea" OR Cuba OR Morocco OR Argentina OR Cyprus OR Hungary OR Honduras OR Oman OR Brunei OR Gabon OR "South Yemen" OR "Dominican Republic" OR Ghana OR "South Africa" OR Madagascar OR Zimbabwe OR Uruguay OR Chile OR "Saudi Arabia" OR Gambia OR Grenada OR Suriname OR Panama OR Romania OR Hyderabad))

Source type: Scholarly Journal

Date limits: After 1 January 1990

**Appendix B. Brief description of included interventions**

| **Level of intervention** | **First author (year), country** | **Brief description of the intervention** |
| --- | --- | --- |
| Individual | Adhikari et al. (2015), Nepal | Adhikari et al. (2015) assessed the impact of a rehabilitation programme on the psychosocial well-being, including depression, anxiety and PTSD, of former child soldiers (verified minors) in Nepal. The programme, organised by the government in partnership with United Nations agencies and NGOs, offered four types of support: (1) formal education support, which included both formal schooling and informal bridging courses; (2) micro-enterprise support, including business training, short courses, and support to start a business; (3) vocational training to develop skills for occupations such as welding, electrical work, and carpentry; and (4) health sector training and education for roles such as health assistants, nurses, and laboratory technicians. In addition, regional psychosocial counsellors provided psychosocial support. |
| Family | Leon-Jurado et al. (2022), Colombia | Leon-Jurado and Maldonado (2022) studied a poverty reduction initiative called "Transformando Mi Futuro" (Transforming My Future, TMF), which aimed to provide victims of armed conflict with tools to optimise the use of their monetary reparations. The programme targeted households registered in the victims' registry that chose to invest their reparations in productive enterprises. The TMF focused on four key components: (i) financial education, (ii) entrepreneurship (technical training), (iii) life skills coaching, and (iv) consumption support. These components were delivered through home visits facilitated by a specially designed app and supported by coaches. The training programme lasted nine months and included 12 visits per household. |
|  | Glass et al. (2017), Democratic Republic of Congo | Glass et al. (2017) evaluated the Pigs for Peace (PFP) intervention, a hybrid programme combining microcredit and productive asset transfer principles in rural Democratic Republic of Congo. In this randomised controlled trial, the intervention group received a female piglet as a productive asset, along with practical training on nutrition and livestock care. Participants also benefited from bi-weekly home visits by trained staff, support for association meetings, and basic health services provided by a local veterinarian. Participants were required to raise the piglet and repay the 'loan' by transferring two piglets from the first litter: one as repayment for the original asset transfer and one to the delayed control group as interest. |
| Community | Kanyangara et al. (2007), Rwanda; Rimé et al. (2011), Rwanda^;^ Brounéus (2010), Rwanda; Caparos et al. (2020), Rwanda | Gacaca courts in Rwanda: Following the 1994 genocide against the Tutsi, in which over one million people (approximately 10% of the population) were killed in three months, the post-genocide government established the Gacaca system. This system consisted of 12,000 courts in every community in the country, made up of people of integrity chosen by the community. The accused were given the opportunity to explain their actions to a judge, survivors and the community. The parties to the conflict were expected to listen to each other's grievances and suffering in order to promote understanding and empathy. Sentences for perpetrators ranged from civil compensation for the least serious crimes to life imprisonment for the most serious. Prisoners who confessed before the trial and asked for forgiveness could receive significant reductions in their sentences. |
|  | Cilliers et al. (2016), Sierra Leone | This intervention was implemented by a non-governmental organisation (NGO) in Sierra Leone known as Fambul Tok. The programme involved training community committees in trauma healing and mediation and encouraging both victims and perpetrators to engage in a community-based truth-telling process. The process culminated in a two-day ceremony where victims told their stories and perpetrators asked for forgiveness. The ceremonies included traditional rituals designed to promote community healing. The ceremony was followed by the planting of a symbolic peace tree and, in some cases, the establishment of community farms. In addition, the NGO facilitated the formation of a Peace Mothers group to address gender-based atrocities committed during the conflict. |
|  | Kang et al. (2020), Rwanda | Kang et al. (2020) conducted a longitudinal study to evaluate the effectiveness of a local peacebuilding intervention known as 'Cows for Peace', which aimed to promote reconciliation between genocide perpetrators (genocidaires) and survivors in Rwanda. The intervention had three key components: (1) a three-day workshop held in the first month, adapted from a cognitive-behavioural programme originally designed for people affected by war and conflict in northern Uganda. The workshop aimed to equip participants with skills to cope with post-traumatic stress and support reconciliation efforts; (2) cell groups, which operated from Months 2 to 22 and consisted of self-led gatherings of genocide survivor dyads who had completed the workshop. These closed groups, which met monthly in local residential areas, were led by a group-appointed leader; and (3) cooperative cow rearing, which took place from months 8 to 22. In this phase, one dyad from each cell group was randomly selected to receive a cow in the eighth month of the programme.  The selected dyads continued to participate in cell groups and engaged in activities related to the care of the cow, including building a cow shed, buying fodder and managing the cow. The benefits of the cow, such as milk, dung (used as fertiliser) and income, were shared between the two households. The cow was raised on the survivor's property and any calf produced was given to the genocidaire. |
|  | Weder et al. (2010), Israel-Palestine | Weder et al. (2010) studied the Parents Circle Families Forum (PCFF), a cross-community peacebuilding project of bereaved families promoting peace, reconciliation and tolerance. Members of the forum have all lost a first-degree relative to violence in the Middle East. The aim of the intervention is therefore to transform feelings of anger and revenge, helplessness and despair into hope and empathy for the pain and needs of others. The programme included i) a telephone dial-in service that facilitated one-to-one telephone conversations between Israelis and Palestinians who did not know each other, b) a school speaker programme in Israeli and Palestinian schools, and c) community lectures around the world. |
| National | Stein et al (2008), South Africa; Kaminer et al (2001), South Africa | South African Truth and Reconciliation Commission (TRC): The main objective of the TRC was to uncover and document human rights violations committed during the apartheid era. As part of the TRC process, many survivors testified either through private statements or at public hearings. Amnesty was granted to perpetrators who offered full disclosure. Two studies have examined the impact of the TRC on mental health and well-being. |
|  | Best et al. (2011), Liberia | Best et al. (2011) conducted a study in Liberia to examine the role of media in facilitating truth-telling and intra-psychic healing. The intervention involved participants interacting with two different media systems. The first system was an interactive Truth and Reconciliation Commission website, which included content from the TRC's activities, such as videos of hearings and official messages. Users were able to contribute content through discussion forum posts, photos and audio recordings. The second system consisted of an interactive computer kiosk, similar to an arcade game, which allowed users to view videos recorded by other Liberians and to record their own videos. This system was designed to provide participants with exposure to a wide range of conflict and reconciliation-related content, as well as the opportunity to contribute their own user-generated videos. The aim of this exposure was to assess "the role of new media in supporting this process of truth-telling and intra-psychic healing by developing participants' sense of self-efficacy - their self-assessed sense of personal competence to deal effectively with stressful situations" (Best et al., 2011, p. 233). |
|  | Qouta et al. (1995), Israel-Palestine | The context of this study is the peace treaty signed between Israel and Palestine in Washington on 13 September 1993. The treaty included international recognition of Palestinian national rights and marked the end of the Intifada, the first Palestinian national uprising. It included a commitment to a cessation of violence. Qouta et al. (1995) aimed to explore the impact of the peace treaty on the psychological well-being of Palestinian children, who, as noted by the authors, "played significant roles in the initiation, planning, and organization of the Intifada" (p. 1198). |
|  | Simancas-Fernandez et al (2022), Colombia | This study focused on the Colombian armed conflict, an internal war that lasted for over 50 years. The civilian population was severely affected, with over 9.9 million victims recognised by 2024, many of whom were displaced from their homes and towns. Colombian Law 1448, enacted in 2011, mandates the provision of reparations to victims through measures such as restitution, compensation, rehabilitation, satisfaction, and guarantees of non-repetition, addressing the individual, collective, material, moral, and symbolic dimensions of harm. |

**Appendix C. List of excluded interventions after full review**

| **Citation** | **Reason for exclusion** |
| --- | --- |
| Aida T (2020) Revisiting suicide rate during wartime: Evidence from the Sri Lankan civil war. PLOS ONE 15(10): e0240487. <https://doi.org/10.1371/journal.pone.0240487> | Wrong intervention |
| Blevins, D., Roca, J. V., & Spencer, T. (2011). Life Guard: Evaluation of an ACT-based workshop to facilitate reintegration of OIF/OEF veterans. *Professional Psychology: Research and Practice, 42*(1), 32–39. [https://doi.org/10.1037/a0022321](https://psycnet.apa.org/doi/10.1037/a0022321) | Wrong intervention |
| Castellano, C., & Everly, G. S., Jr (2010). Large group intervention for military reintegration: peer support & Yellow Ribbon enhancements. *International journal of emergency mental health*, *12*(3), 171–177. | Wrong study design |
| Chase, R., Doney, A., Sivayogan, S., Ariyaratne, V., Satkunanayagam, P., & Swaminathan, A. (1999). Mental health initiatives as peace initiatives in Sri Lankan schoolchildren affected by armed conflict. *Medicine, conflict, and survival*, *15*(4), 379–393. https://doi.org/10.1080/13623699908409479 | Wrong outcome |
| Chauvin, L., Mugaju, J., & Comlavi, J. (1998). Evaluation of the psychosocial trauma recovery programme in Rwanda. *Evaluation and Program Planning, 21*(4), 385–392. [https://doi.org/10.1016/S0149-7189(98)00034-2](https://psycnet.apa.org/doi/10.1016/S0149-7189(98)00034-2) | Wrong intervention |
| Corboz, J., Siddiq, W., Hemat, O., Chirwa, E. D., & Jewkes, R. (2019). What works to prevent violence against children in Afghanistan? Findings of an interrupted time series evaluation of a school-based peace education and community social norms change intervention in Afghanistan. *PloS one*, *14*(8), e0220614. https://doi.org/10.1371/journal.pone.0220614 | Wrong intervention |
| De Tezanos-Pinto, P., Mazziotta, A., & Feuchte, F. (2017). Intergroup contact and reconciliation among Liberian refugees: A multilevel analysis in a multiple groups setting. *Peace and Conflict: Journal of Peace Psychology, 23*(3), 228–238. [https://doi.org/10.1037/pac0000251](https://psycnet.apa.org/doi/10.1037/pac0000251) | Wrong intervention |
| Garzón-Velandia, D. C., Rozo, M. M., & Blanco, A. (2020). From war to life: Psychological well-being in adult victims of child recruitment. *Peace and Conflict: Journal of Peace Psychology, 26*(3), 293–302. [https://doi.org/10.1037/pac0000449](https://psycnet.apa.org/doi/10.1037/pac0000449) | Wrong study design |
| Glass N, Perrin NA, Kohli A, Remy MM (2014) Livestock/Animal Assets Buffer the Impact of Conflict-Related Traumatic Events on Mental Health Symptoms for Rural Women. PLOS ONE 9(11): e111708. <https://doi.org/10.1371/journal.pone.0111708> | Wrong intervention |
| Jansen, S., Nsabimana, E., Kagaba, M., Mutabaruka, J., Rutembesa, E., Slegh, H., Mihigo, B., Mahwa, A., Ruratotoye, B., & Haile, Z. (2022). A clustered randomized controlled trial to assess whether Living Peace Intervention (LPint) reduces domestic violence and its consequences among families of targeted men in Eastern Democratic Republic of the Congo (DRC): Design and methods. *Evaluation and program planning*, *95*, 102154. https://doi.org/10.1016/j.evalprogplan.2022.102154 | Wrong intervention |
| Johnson, L. R., Mudryk, E., Kibanja, G. M., Drescher, C. F., & Malindi, M. (2023). Is positive youth development a pathway for sustaining peace in Tanzania? Predicting peace attitudes among adolescents. *Peace and Conflict: Journal of Peace Psychology, 29*(3), 225–234. [https://doi.org/10.1037/pac0000673](https://psycnet.apa.org/doi/10.1037/pac0000673) | Wrong intervention |
| Kira, I. A., Lewandowski, L., Templin, T., Ramaswamy, V., Ozkan, B., Hammad, A., & Mohanesh, J. (2006). The Mental Health Effects of Retributive Justice: The Case of Iraqi Refugees. *Journal of Muslim Mental Health*, *1*(2), 145–169. https://doi.org/10.1080/15564900600980756 | Wrong population |
| Kira, I. A., Lewandowski, L. A., Templin, T. N., Ramaswamy, V., Ozkan, B., & Mohanesh, J. (2009). The Effects of Post-Retribution Inter-Group Forgiveness: The Case of Iraqi Refugees. *Peace and Conflict: Journal of Peace Psychology*, *15*(4), 385–413. https://doi.org/10.1080/10781910903158669 | Wrong intervention |
| Laplante, L. J., & Holguin, M. R. (2006). The Peruvian Truth Commission's mental health reparations: empowering survivors of political violence to impact public health policy. *Health and human rights*, *9*(2), 136–163. | Wrong outcome |
| Mendeloff, D. (2009). Trauma and Vengeance: Assessing the Psychological and Emotional Effects of Post-Conflict Justice. *Human Rights Quarterly*, *31*(3), 592–623. http://www.jstor.org/stable/40389961 | Wrong study design |
| Morley, C. A., & Kohrt, B. A. (2013). Impact of Peer Support on PTSD, Hope, and Functional Impairment: A Mixed-Methods Study of Child Soldiers in Nepal. *Journal of Aggression, Maltreatment & Trauma*, *22*(7), 714–734. https://doi.org/10.1080/10926771.2013.813882 | Wrong intervention |
| Paphitis, S., Akilu, F., Chilambo, N., Iruayenama, A., Samaroo, X., Mustapha, A., Goldsmith, K., Ismail, O., Slovak, P., Ikpe, E., Smith, P., Patel, P., Sullivan, R., Abas, M., & Olonisakin, F. (2023). Toward an integrated approach for mental health and psychosocial support and peacebuilding in North-East Nigeria: programme description and preliminary outcomes from 'Counselling on Wheels'. *BJPsych open*, *9*(6), e190. https://doi.org/10.1192/bjo.2023.575 | Wrong intervention |
| Tanyel, S. S., & Kıralp, F. S. Ş. (2021). Tolerance for sustainable peace culture in a divided society: The effect of peace education on tolerance tendency and human values. *Social Indicators Research, 156*(1), 223–246. [https://doi.org/10.1007/s11205-021-02630-w](https://psycnet.apa.org/doi/10.1007/s11205-021-02630-w) | Wrong outcome |
| Toussaint, L. L., Peddle, N., Cheadle, A., Sellu, A., & Luskin, F. (2010). Striving for peace through forgiveness in Sierra Leone: Effectiveness of a psychoeducational forgiveness intervention. In A. Kalayjian & D. Eugene (Eds.), *Mass trauma and emotional healing around the world: Rituals and practices for resilience and meaning-making, Vol. 2. Human-made disasters* (pp. 251–267). Praeger/ABC-CLIO. | Wrong publication type |
| Ubillos-Landa, S., Puente-Martínez, A., Arias-Rodríguez, G., Gracia-Leiva, M., & González-Castro, J. L. (2019). Coping Strategies Used by Female Victims of the Colombian Armed Conflict: The Women in the Colombian Conflict (MUCOCO) Program. *Sage Open*, *9*(4). <https://doi.org/10.1177/2158244019894072> (Original work published 2019) | Wrong intervention |
| Abu Hamad, B., Jones, N., Abuhamad, S., Baird, S., & Oakley, E. (2025). Can social protection contribute to social connectedness in contexts of forced displacement and crisis? Lessons from Jordan's labelled cash transfer for education [Article]. World Development, 188, Article 106886. <https://doi.org/10.1016/j.worlddev.2024.106886> | Wrong intervention |
| Alberio, M., Otálora-Buitrago, A., Acevedo, J. A. R., & Vergara, R. (2025). Proyecto Utopía: Colombian Initiative for Peace and the Defense of Rurality in Times of Post-Agreement [Article]. Canadian Journal of Nonprofit and Social Economy Research, 16(2), 79-91. <https://doi.org/10.29173/cjnser808> | Wrong publication type |
| Duman, Y., Meier, J., & Marzouk, H. (2024). Beyond Survival: Transformative Impacts of Integrating Mental Health and Livelihood Support in Conflict Zones [Article]. Journal of Peacebuilding and Development, 19(2-3), 158-176. <https://doi.org/10.1177/15423166241293844> | Wrong intervention |
| Habimana, S., Freeman, K., Biracyaza, E., Lister, Z., & Montgomery, S. (2026). Promoting social cohesion, compassion and forgiveness among Rwandan survivors and perpetrators through community resiliency model training: a longitudinal randomized comparison study. European Journal of Psychotraumatology, 17(1), 2621634. <https://doi.org/https://dx.doi.org/10.1080/20008066.2026.2621634> | Wrong intervention |
| Jansen, S., Niyonzima, J. B., Gerbarg, P., Brown, R. P., Nsengiyumva, A., Niyonsenga, J., & Nsabimana, E. (2024). Evaluating effects of community-based social healing model on Ubuntu, mental health and psychosocial functioning in post-genocide Rwanda: protocol for cluster randomized control trial. Trials [Electronic Resource], 25(1), 773. <https://doi.org/https://dx.doi.org/10.1186/s13063-024-08632-6> | Wrong intervention |
| Kapshuk, Y. (2026). The moderating role of conflict intensity in peace education [Article]. Peace and Conflict. <https://doi.org/10.1037/pac0000821> | Wrong outcome |
| Khanal, D., Sitaula, S., Koirala, P., Gautam, K., & Koirala, S. (2024). Outcomes of integrating livelihood into mental health and psychosocial support program among survivors of torture: A mixed-method study from western Nepal. Torture, 34(2), 41-51. <https://doi.org/https://dx.doi.org/10.7146/torture.v34i2.140430> | Wrong intervention |
| Lesley, E., Bonumwezi, J. L., Taing, S., Marea, C., Balasubramanian, S., & Minasovi, G. (2024). Genocide as communitarian breakdown: Interventions for relational healing and individual wellbeing in Rwanda and Cambodia [Behavior Disorders & Antisocial Behavior 3230]. SSM Mental Health, 6. <https://doi.org/https://dx.doi.org/10.1016/j.ssmmh.2024.100328> | Wrong publication type |
| Rocha-Payares, A. S., Palacio Sanudo, J. E., Macias, M. A., & Zapata Ospino, M. P. (2025). Design and effect of ludic-educational instruments on forgiveness in victims of the armed conflict in the Colombian caribbean region [Tests & Testing 2220 Group & Interpersonal Processes 3020]. Diseno y efecto de herramientas ludico-pedagogicas sobre el perdon en personas victimas del conflicto armado en el caribe colombiano., 24, 1-14 | Wrong outcome |
| Velez, G., & Idrobo, F. (2024). From forgiveness and reconciliation to social capital and psychosocial well-being: An evaluation of a multisite intervention in Colombia [Community & Social Services 3373]. European Journal of Social Psychology, 54(5), 1050-1064. <https://doi.org/https://dx.doi.org/10.1002/ejsp.3033> | Wrong outcome |
